# A comprehensive overview of substrate specificity of glycoside hydrolases and transporters in the small intestine: “A gut feeling”

**DOI:** 10.1007/s00018-020-03564-1

**Published:** 2020-06-06

**Authors:** Hidde Elferink, Jeroen P. J. Bruekers, Gerrit H. Veeneman, Thomas J. Boltje

**Affiliations:** 1 Institute for Molecules and Materials, Radboud University, Heyendaalseweg 135, 6525 Nijmegen, The Netherlands; 2 PharmaCytics B.V., Noviotech Campus, Nijmegen, The Netherlands

## Abstract

The human body is able to process and transport a complex variety of carbohydrates, unlocking their nutritional value as energy source or as important building block. The endogenous glycosyl hydrolases (glycosidases) and glycosyl transporter proteins located in the enterocytes of the small intestine play a crucial role in this process and digest and/or transport nutritional sugars based on their structural features. It is for these reasons that glycosidases and glycosyl transporters are interesting therapeutic targets to combat sugar related diseases (such as diabetes) or to improve drug delivery. In this review we provide a detailed overview focused on the molecular structure of the substrates involved as a solid base to start from and to fuel research in the area of therapeutics and diagnostics.

In most diets, carbohydrates constitute a major source of energy in the form of digestible oligo- and disaccharides. For example, amylose (*α*-1,4-linked glucose) and amylopectin (α-1,6 branched *α*-1,4-linked glucose) are components of starches and an important source of glucose. Disaccharides such as sucrose (*α*-Glc-1 → 2-*β*-Fruc), lactose (*β*-Gal-1 → 4-Glc) and trehalose (*α*-Glc-1 → 1-*α*-Glc) are precursors for fructose, galactose and glucose, respectively. To unlock the nutritional value of these molecules, the body expresses enzymes to hydrolyze the oligo- and disaccharides into their respective monosaccharide constituents followed by uptake via a series of monosaccharide transporters [1].

Upstream of intestinal digestion, large α-linked polysaccharides such as amylose are degraded to disaccharides (e.g. maltose) and oligosaccharides by salivary- and pancreatic amylase [2]. In addition, a small amount is hydrolyzed in the stomach [3]. As these processes upstream of the small intestine produce negligible quantities of transportable sugars, they are beyond the scope of this review. The bulk of oligo- and disaccharide digestion and uptake occurs in the small intestine by the action of membrane bound glycosyl hydrolases present in the brush border of the jejunum (Fig. 1). The breakdown of starch oligosaccharides is carried out by enzymes capable of cleaving the *α*-1,6-Glc and *α*-1,4-Glc linkages such as the maltase-glucoamylase complex (MGAM) and sucrase isomaltase (SI). In addition, SI is also capable of cleaving sucrose into glucose and fructose. Lactose is converted by lactase-phlorizin hydrolase (LPH) yielding galactose and glucose. Trehalose, a disaccharides found in mushrooms, can be cleaved by trehalase (TREH) into two molecules of glucose. In addition to membrane bound glycosyl hydrolases, enterocytes also express intracellular human cytosolic *β*-glucosidase (hCBG), a broad specificity *β*-glycosidase enzyme.

**Fig. 1:**
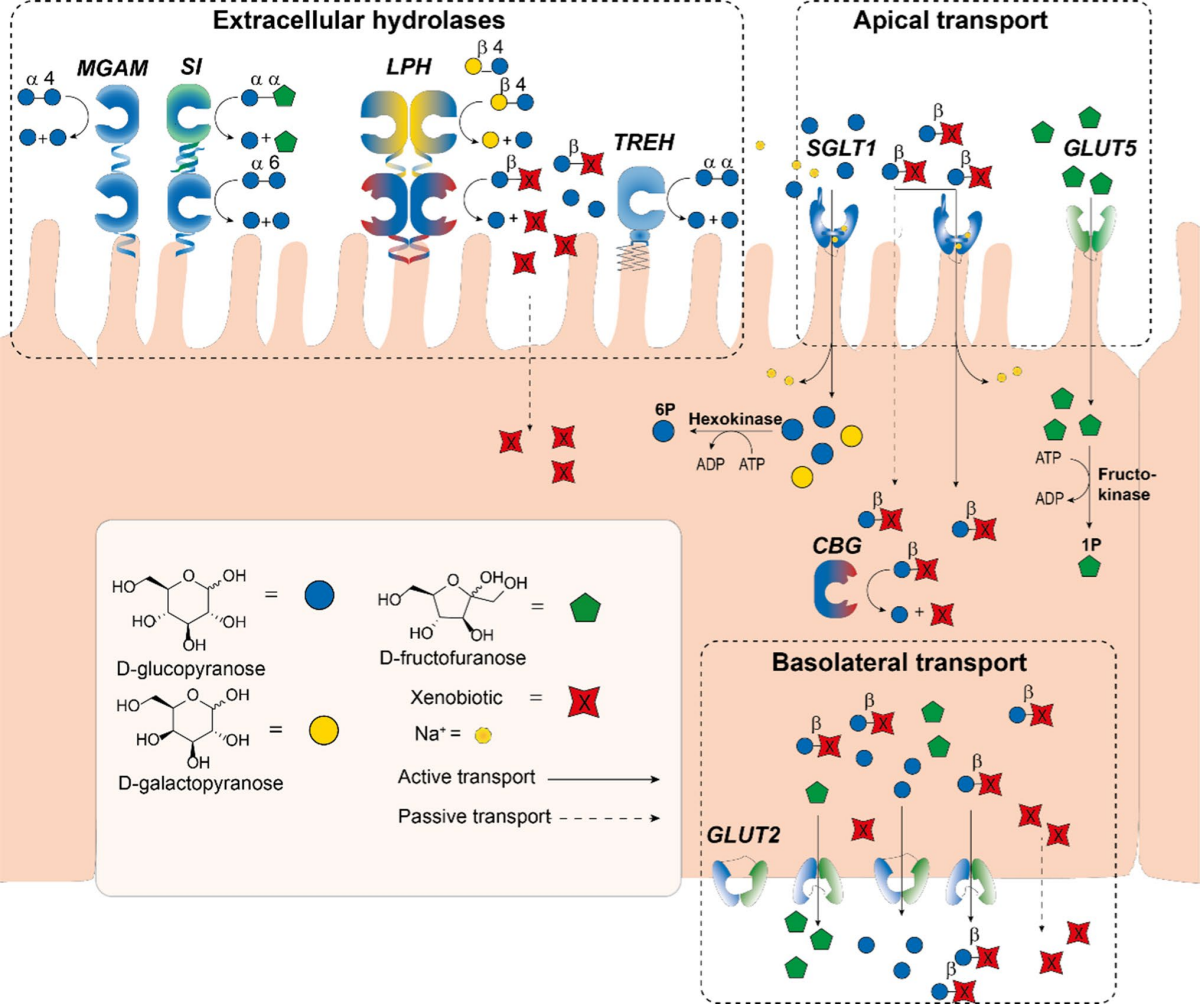
Schematic overview of carbohydrate hydrolysis and transport in the enterocytes of the small intestine. Dietary saccharides and digested to produce monosaccharides that enter the enterocytes at the apical side of the enterocyte (top face) and are released at the basolateral side. MGAM (maltase glucoamylase), SI (sucrase isomaltase), LPH (lactase phlorizin hydrolase), TREH (trehalose), CBG (cytosolic *β*-glucosidase), SGLT1 (sodium dependent glucose transporter 1), GLUT5 (glucose transporter 5), GLUT2 (glucose transporter 2)

The monosaccharides produced by glycosylhydrolases in the small intestine traverse the enterocytes lining the jejunum with the assistance of a number of monosaccharides transporters. Glucose and galactose are mainly taken up by SGLT1, a symport transporter of these monosaccharides and two sodium ions. Fructose is passively transported independent of sodium via facilitated transport by the action of GLUT5. SGLT1 and GLUT5 represent the major tools for enterocytes to enable monosaccharide uptake from the apical side. Subsequent basolateral excretion of monosaccharides into the bloodstream is facilitated by GLUT2.

The hydrolysis and uptake of carbohydrates is adaptable and responds to changes in the need for nutrients and their availability [4]. For example, increased glucose absorption is observed during pregnancy and lactation [5] or after surgical resection of the intestine [6]. The importance of a healthy interplay between intestinal glycosyl hydrolase activity and monosaccharide transport is further illustrated by diseases resulting from the altered activity of intestinal hydrolases and transporters. The most common disorder in this respect is lactose intolerance, a drop in lactase activity causing a build-up of lactose [7]. High lactose levels lead to the retention of water in the lumen resulting in diarrhea and other discomforts.

Hence, the processing and uptake of dietary carbohydrates represents an important mechanism to take up energy and is crucial in health and disease. The observation that glycosylated flavonoids are better taken up compared to their non-glycosylated variants indicates that glycoconjugation and related hydrolysis and transport mechanisms may be exploited for improving oral drug uptake. Furthermore, modulating carbohydrate processing and uptake is an important therapeutic avenue to treat diabetes. In this review, we aim to summarize the current state of knowledge regarding the hydrolysis of oligo- and disaccharides and transport of the resulting monosaccharides at the molecular level. To this end, we will discuss the structure of the hydrolases and transporters involved as well as the scope of modifications of the monosaccharides that are tolerated. This information will be crucial to advance therapeutic and diagnostic efforts directed at the intestinal carbohydrate metabolism.

## General introduction

### Glycosidase activity in the small intestinal brush border

See Table 1.

**Table 1:**
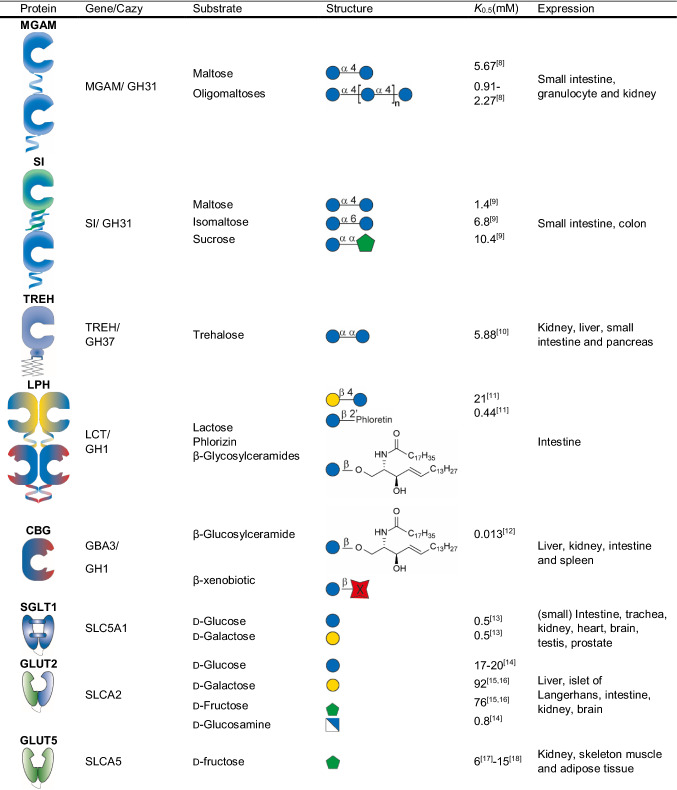
Overview of human glycosidases and monosaccharide transporters and in the small intestine

## Glycosyl hydrolases in the small intestine

**Fig. 2:**
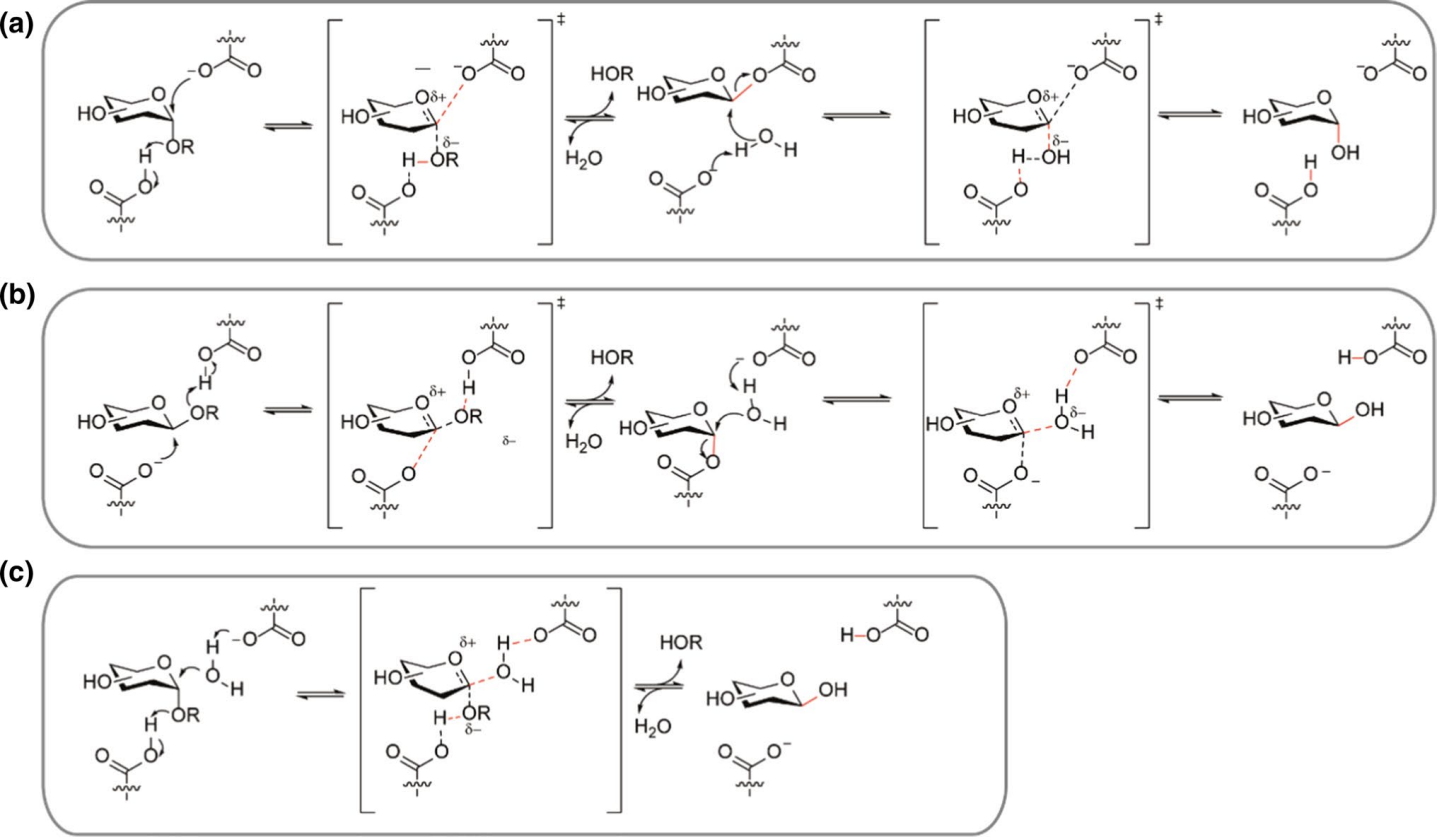
Overview of Koshland’s mechanism of glycoside hydrolysis in human intestinal glycosidases. **a** Retaining mechanism for *α*-glucosidases SI and MGAM [22, 25]. **b** Retaining mechanism of CBG and LPH in the hydrolysis of *β*-conjugated glycosides [22, 23, 25, 26]. **c** Inverting mechanism of the *α*-glycosidase TREH [22, 25, 27]. Lines in red indicate the formation of new bonds

The small intestinal brush border membrane and the cytosol of the enterocytes, contain glycosidases capable of hydrolyzing various ingested saccharides converting non-absorbable di- and oligosaccharides into nutritional monosaccharides [19]. The five endogenous glycosidases (or glycosyl hydrolases) involved in this process are MGAM, SI, LPH, TREH and CBG (Fig. 1). The glycosidases are part of a family of enzymes that catalyze the hydrolysis of the glycosidic linkage of (oligo)-saccharides or the glycosidic linkage between a carbohydrate (glycone) and a non-carbohydrate moiety (aglycone) [20]. The hydrolysis of the glycosidic bond is catalyzed by the carboxylic acid moieties of two amino acid residues of the enzyme: a general acid (proton donor) and a nucleophile/base. Depending on the arrangement of these groups hydrolysis of the glycosidic bond occurs with retention or inversion of the anomeric configuration [21–23]. MGAM and SI hydrolyze α-glucosides with retention of configuration (Fig. 2a) and LPH and CBG hydrolyze *β*-glycosides with retention of configuration (Fig. 2b). In contrast, TREH digests *α*,*α*-trehalose with inversion of anomeric stereochemistry (Fig. 2c). The endogenous glycosidases are differentially expressed over the length of the small intestine. For example, MGAM is increasingly expressed along the intestine with the highest level in the distal ileum [24]. SI and LPH are mostly expressed in the jejunum with lower expression levels towards the proximal and distal ends of the intestine, whereas the spatial expression of TREH has not been reported yet [24]. Glycosidase deficiency leads to the inability to digest certain sugars causing symptoms such as chronic diarrhoea and malabsorption. The pathology or these deficiencies will be discussed briefly in every section.

A classification has been made into glycosyl hydrolase (GH) families (145 families to date) depending on the amino acid sequence similarities and these can be further classified into groups (reported as clans) by similarities in folds and catalytic mechanism (see the Carbohydrate Active enZYme or CAZY data set https://www.cazy.org) [20, 22]. In addition, in this review the sub-site nomenclature proposed by Davies et al. for glycosyl hydrolase binding sites is adopted, where *n* represents the reducing end of the carbohydrate chain, − *n* represents the non-reducing end and cleavage occurs between the − 1 and + 1 binding sites [28].

### Maltase-glucoamylase as intestinal glycosidase

The maltase-glucoamylase (MGAM, EC 3.2.1.20/3) complex accounts for all small intestinal glucoamylase activity, 20% of the maltase activity and a lesser amount of the isomaltase activity [29]. MGAM is expressed in the small intestinal brush border, representing about 2% of the brush border membrane proteins. In addition to the small intestine, MGAM is present in the renal tubular brush border membrane (Table 1). It is comprised of two subunits: the *N*-terminal subunit which hydrolyzes maltose (referred to as maltase) and the *C*-terminal subunit, that is able to hydrolyse longer oligomaltose substrates (als known as glucoamylase). It should be noted that although MGAM has a high activity for starch related oligomaltoses, at mealtime concentrations it experiences substrate inhibition. Instead, studies have shown that at high oligomaltose concentration sucrose-isomaltase contributes to most of the *α*-glycosidase activity [30]. MGAM deficiency disease in infant children leads to the inability to digest short chain oligomaltoses and results in chronic diarrhoea but not necessarily in malabsorption. The symptoms can be treated by avoiding starch and starch-containing products in the diet [31]. Though the exact origin of MGAM deficiency remains unclear studies have shown it is likely not genetic [32]. Specific information about folds/domains and the crystal structure (*N*-terminal 3L4X [33], 2QMJ [34] and *C*-Terminal 3TON [8] and 3TOP [8]) can be found in studies by Rose and co-workers [34] and Ren et al*.* [8]. Various splice forms of ctMGAM exist in mammals and can be used for hydrolysis studies. The splice forms N2 and N20 from mice are often used as a model for human MGAM (hMGAM) as they have a 80% sequence identity [35]. Critically, the affinity of substrates tested may differ from the hMGAM.

**Fig. 3:**
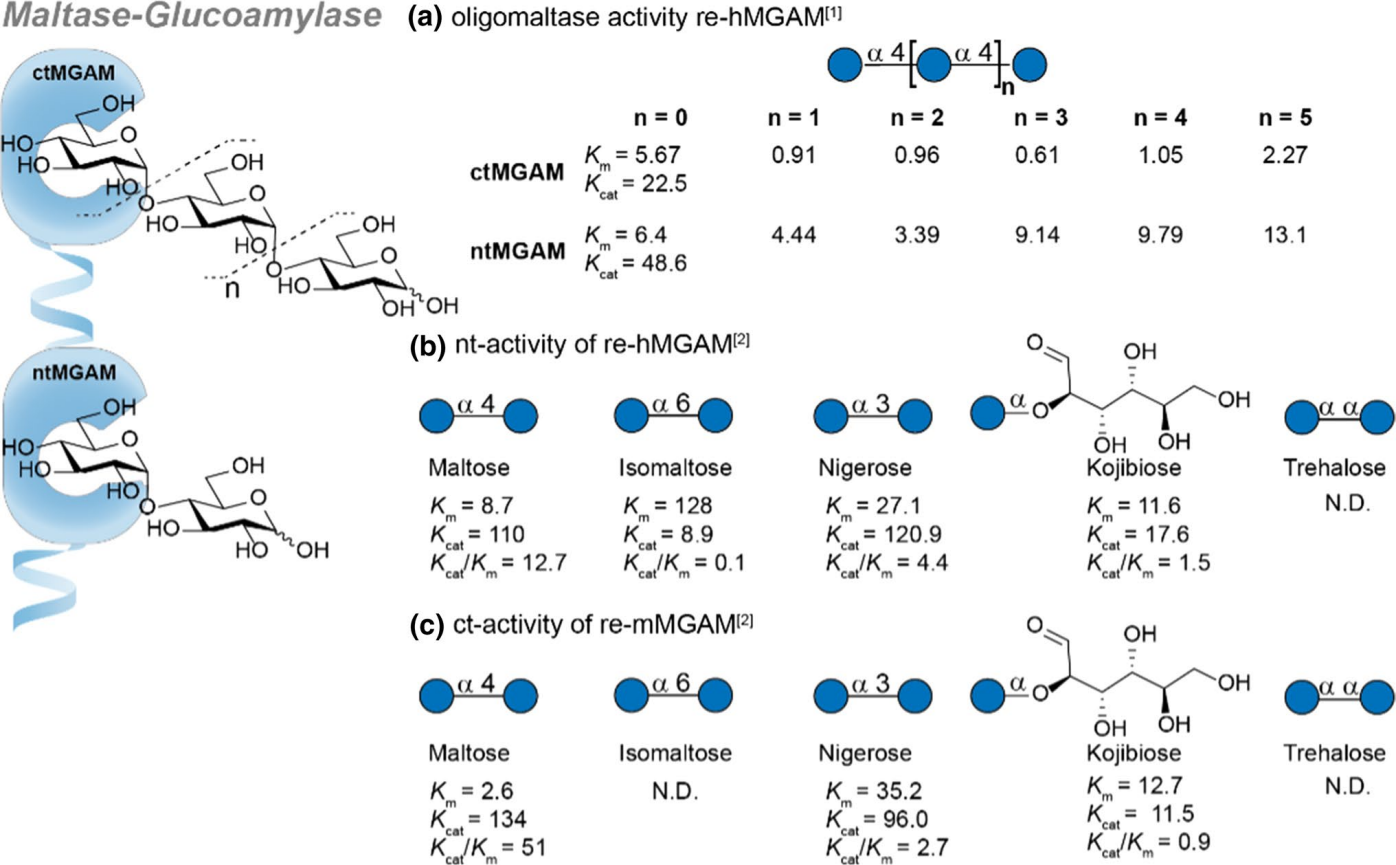
Overview of the MGAM substrate affinity. **a** Substrate affinity of recombinant human MGAM *C*-terminal (ctMGAM) and *N*-terminal (ntMGAM) subunits towards oligomaltoses. **b** Overview of the nt-activity of re-hMGAM towards disaccharides. **c** Summarizes identical activity trend for recombinant mouse *C*-terminal MGAM (re-mMGAM). *K*_m_ (mM) and *K*_cat_ in (s^−1^). References used: ref 1 [8], ref 2 [36]

The wild-type enzyme has a preference for *α*-1,4 substituted oligo-glucosides (oligomaltose) and can hydrolyze increasing lengths up to glucohexaose efficiently. The regiochemistry is important as *α*-1,6-glucosidic bonds are hydrolysed at only 2% of the rate of *α*-1,4-glucosidic linkages. In addition, there is some *α*-1,2- and *α*-1,3-glycosidase activity present whilst sucrose (*α*-d-glucopyranosyl-(1 → 2)-*β*-d-fructofuranoside) and *β*-1,4 and *β*-1,6 glucosidic linkages are not hydrolysed [29, 37–39]. The substrate affinity and related activity of MGAM is summarized in Fig. 3. Data from the research groups of Hamaker [36] and Shen [8] are used for comparison. The group of Shen was first to report the crystal structure and substrate specificity for the human ctMGAM and compared this to recombinant human ntMGAM (Fig. 3a). The trends observed in substrate affinity of the *C*-terminal subunit clearly indicate the preference for longer chain maltose oligosaccharides with increasing affinity up to maltopentaose (Fig. 3a, *n* = 3). Critically, the crystal structure showed that the ctMGAM had additional + 2 and + 3 binding subsites which may explain the preference of ctMGAM for long chain sugars. In contrast, the ntMGAM subunit shows a preference for shorter oligosaccharides. The activity towards disaccharides was studied on *C*-terminal re-mMGAM and *N*-terminal re-hMGAM by Hamaker and coworkers (Fig. 3b, c). Remarkably, nigerose and kojibiose were accepted in both subunits as substrates whereas no hydrolytic activity towards trehalose was observed. In addition, unexpected sucrase activity was found in the recombinant mouse ctMGAM used. Though interesting, the contribution to total sucrase activity is expected to be marginal since the amount of MGAM is forty times lower than SI in the small intestine. These important studies performed have fuelled numerous related research towards inhibitors of the MGAM complex (see Fig. 16, acarbose). Though MGAM is interesting as drug target against diabetes 2 and obesity, an elaborate discussion about inhibitory activities is beyond the scope of this review.

### Sucrase-isomaltase as intestinal glycosidase

Sucrase isomaltase (SI, EC 3.2.1.48) accounts for approximately 80% of the overall intestinal maltase activity and almost all sucrase activity present in the intestine [40]. SI is one of the major proteins in the small intestinal brush border membrane in terms of abundance, making up about 8–10% of the proteins expressed [41]. Though abundantly present in mammals, SI is not significantly expressed until the weaning period after which expression levels stabilize during adult life [19]. The protein consists similar to MGAM of two catalytic subunits: the *C*-terminal subunit (sucrase) that hydrolyses sucrose (also known as saccharose or *α*-d-glucopyranosyl-(1 → 2)-*β*-d-fructofuranoside) to d-glucose and d-fructose. The d-glucose part of the molecule acts as glycon in this reaction. The *N*-terminal subunit (isomaltase) is mainly involved in the hydrolysis of isomaltose (*α*-d-glucopyranosyl-(1 → 6)-*α*-d-glucopyranoside) into two *α*-d-glucose molecules. Both subunits of SI hydrolyse the substrates via a retaining mechanism (Fig. 2a). In contrast to MGAM, the subunits of SI are connected via a non-covalent interaction which is a result of the proteolytic cleavage of the pro-protein in situ [42]. SI also differs from MGAM as it has a low activity towards oligosaccharides [19, 29]. Interestingly, Naim and coworkers showed by selectively mutating and studying the separate catalytic sites that the sum of maltase activities of the individual subunits is higher than the maltase activity of the wild type enzyme. The glucose produced is able to inhibit SI activity both competitively, at the active site, and uncompetitively, via allosteric interactions [43]. The inhibitory effect observed is analogous to the effect observed in MGAM and is likely a regulatory mechanism to prevent excess glucose release in the small intestine. Congenital SI-deficiency (CSID) is an autosomal recessive disease that stems from mutations in the SI-complex. CSID leads to a total absence of sucrase activity whereas the isomaltase activity varies from low to normal depending on the phenotype [44]. Symptoms are vomiting, diarrhea and can in extremes cases lead to dehydration, developmental retardation and muscular hypothonia after sugar ingestion. Treatment in these cases is a lifelong sucrose restriction. For a detailed overview of CSID we refer to a review by Naim et al*.* [45]. Although the crystal structure of sucrase has not been obtained yet, the isomaltase-subunit of SI (3LPP) [34] has been crystallized. For structural comparison, Gericke et al.[43] modelled the sucrase sub-unit and compared it to the aforementioned crystal structure of isomaltase. The comparison showed, the active sites are likely to be very similar although ultimately a crystal structure of ctSI is needed to confirm this.

**Fig. 4:**
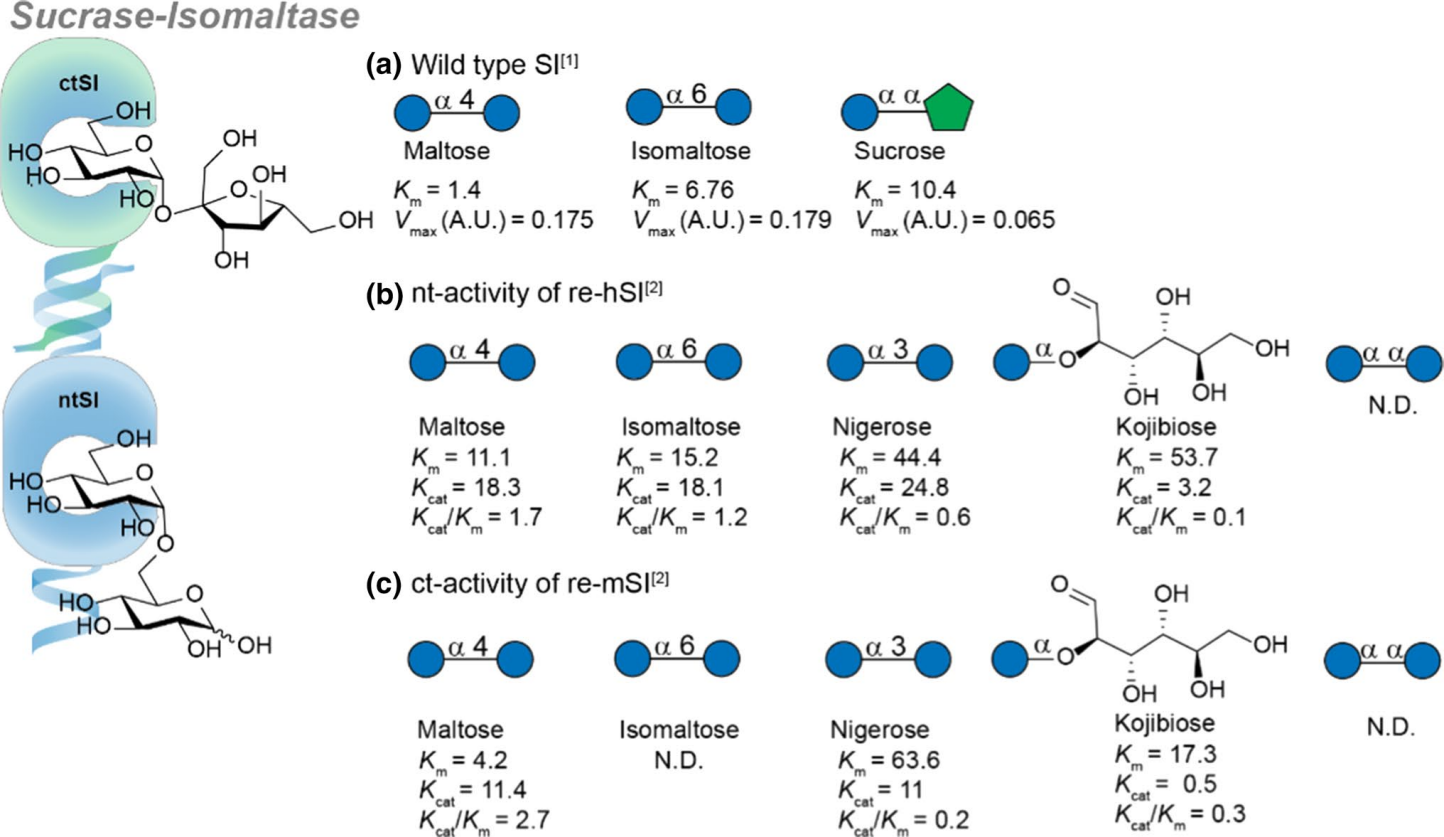
Substrate specificity of sucrase-isomaltase (SI). **a** The affinity of substrates in wild-type SI. **b** The N-terminal activity of human SI (hSI). **c** The C-terminal activity of mouse SI (mSI). Values are reported as: *K*_m_ (mM) and *K*_cat_ in (s^−1^). References used: ref 1 [9], ref 2 [36]. N.D. not detected

Figure 4a shows the combined activity of both subunits of wild-type hSI. As previously mentioned, the sucrase subunit contains practically all sucrase activity present in the small intestine. In addition, hSI can hydrolyze both *α*-substituted gluco-type disaccharides maltose and isomaltose with a comparable *V*_max_, though maltose has a lower *K*_m_. Specific activity of both subunits was determined by recombinant expression of the *N*-terminus based on human cDNA (re-hSI, Fig. 4b) and the *C*-terminus based on mouse cDNA (re-mSI, Fig. 4c). Interestingly, both subunits can hydrolyze maltose, nigerose and kojibiose but all isomaltase activity is present in the *N*-terminal subunit. Analogous to MGAM, neither subunit can hydrolyze trehalose.

### Trehalase activity in the small intestine

Trehalase (TREH, EC 3.2.1.28) is the endogenous glycosidase responsible for the hydrolysis of trehalose (*α*-d-glucopyranosyl-*α*-d-glucopyranoside), which is a non-reducing disaccharide of 1,1′-*α*,*α*’-linked glucose moieties [19]. TREH makes up about 0.1–0.3% of total protein in the intestinal brush border membrane [29] and is also expressed in the renal tubular epithelium [19, 46, 47]. As trehalose is mainly found in funghi, yeast and insects it is no surprise that most known trehalases are found in fungi, insects and bacteria [19, 48, 49]. Trehalase has an inverting mechanism relying on an acid catalysed (Asp^312^) departure of the leaving group followed by a base catalysed (Glu^496^) nucleophilic attack of water, yielding equimolar amounts of *α*- and *β*-d-glucose from trehalose (Fig. 2c) [19, 27, 50, 51]. Though the expression of trehalase in humans is limited, TREH deficiency can lead to diarrhea in combination with foods high in trehalose such as mushrooms [52].

**Fig. 5:**
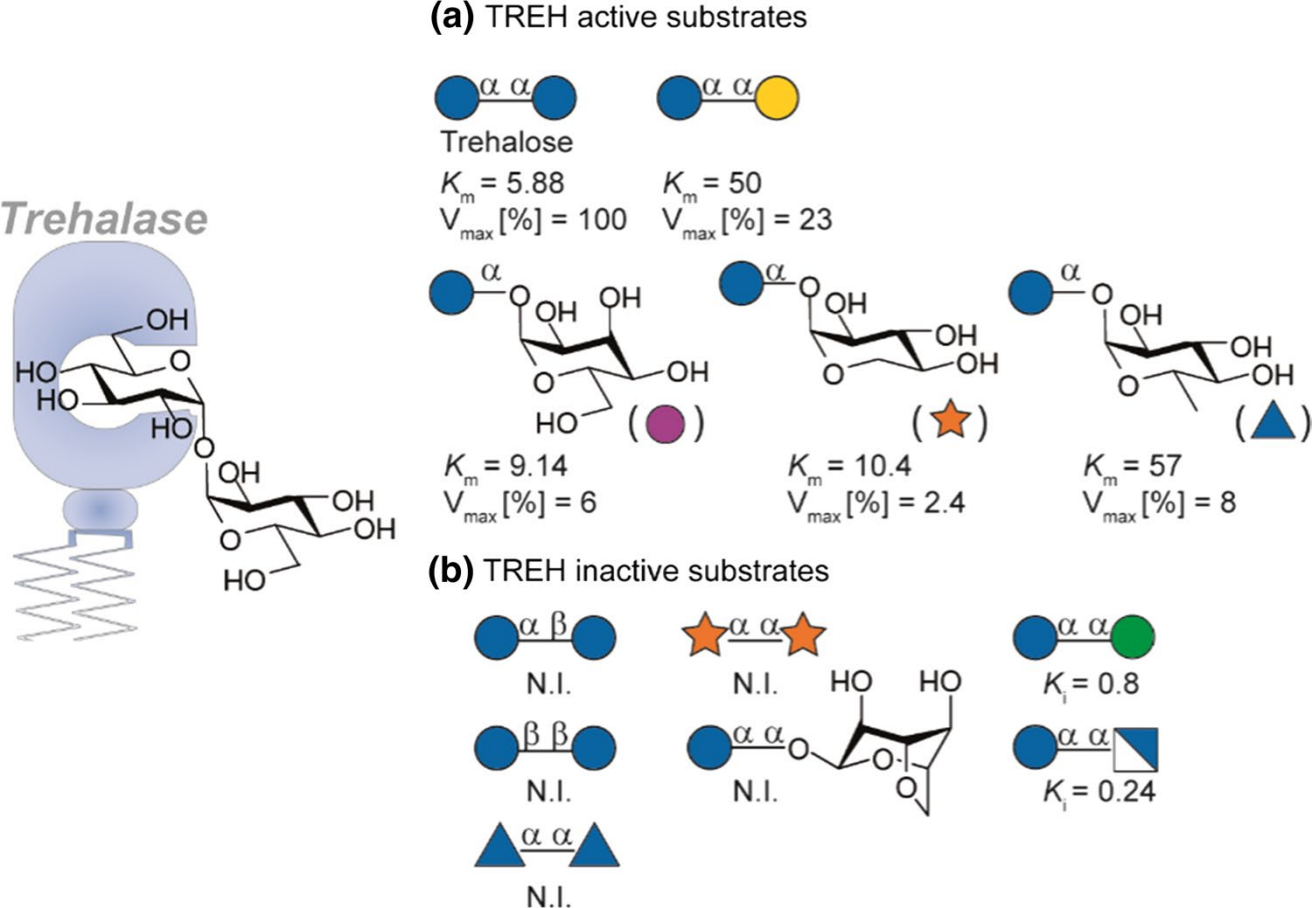
Relative substrate affinity of human TREH. **a** Substrates cleaved by TREH and **b** substrates not cleaved by TREH. *K*_m_ and *K*_i_ are expressed in mM, the *V*_max_ is expressed relative to trehalose (100%) [10]. N.I (no interaction)

Next to trehalose, epimers such as *α*,*α-*glc-gal, *α*,*α-*glc-allose, *α*,*α-*glc-xylose and 6′-deoxytrehalose are hydrolysed by human trehalase (Fig. 5a) albeit with lower efficiency compared to trehalose. In addition, C-2 modification are not tolerated as *α*,*α*-glc-man and *α*,*α*-glc-glcN showed competitive inhibition of TREH. The *α*-glycosidic linkage seems essential for activity and recognition as related *α*,*β*- or *β*,*β*-trehalosides are not recognized. Furthermore, *α*,*α*-analogues lacking a glucoside do not show affinity for TREH, suggesting glucose is the glycon in asymmetric active substrates. The aglycone may not need to be a carbohydrate as studies using rabbit kidney trehalose is showed enzymatic hydrolysis of both *α*-d-glucosyl fluoride (35.6 μmol min^−1^ mg^−1^) and *β*-d-glucosyl fluoride (0.51 μmol min^−1^ mg^−1^), the former even faster than α,α-trehalose (14.3 μmol min^−1^ mg^−1^) [19, 53]. In contrast, trehalase is competitively inhibited by phlorizin, sucrose and TRIS [tris(hydroxymethyl)aminomethane)] [47, 54]. For further insight in the role of trehalose and trehalase in organisms we refer to a review from Carroll and coworkers [55].

### The function of lactase-phlorizin hydrolase in the small intestine

Lactase-phlorizin hydrolase (LPH, EC 3.2.1.23/62) is the major *β*-glycosidase of the intestinal microvillus membrane and accounts for the hydrolysis of lactose and most *β*-glycosylated xenobiotics (e.g. phlorizin and glycosylated flavonoids, Fig. 7a) [11]. LPH is the most important glycosidase in post-natal life in mammals, since the main ingested carbohydrate during this period is lactose. In most mammals its expression levels decrease as the organism grows older and the significance of lactose in daily nutrient ingestion diminishes [19]. The ultimate absence of LPH results in lactose intolerance and this is the most common intestinal disorder associated with decreased activity of glycosidases [56]. Lactose intolerance is classified in four different types in which the extreme case is the autosomal recessive disorder: congenital lactase deficiency (CLD). The very low lactase activity present is causes severe symptoms like watery diarrhoea meteorism and malnutrition. This is life threatening for new borns and needs immediate removal of lactose from the diet and displacement with milk-substitutes. For a comprehensive overview of LPH-pathology we refer to a recent review by Naim and coworkers [57]. Analogous to MGAM and SI, LPH contains two catalytic sites present but the larger enzyme is present as a dimer in the apical membrane (see Fig. 1). Interestingly, the enzyme is synthesized as monomeric pro-LPH molecule with four domains (I–IV). Domains I and II are removed during transport and sorting to the apical membrane leaving domain III and IV which contain the phlorizin-hydrolase and lactase activity, respectively [58]. The lactase sub-site releases *β*-d-galactose and *β*-d-glucose from lactose and has a broad substrate specificity to tolerate various glycosides [59]. Lactase activity is competitively inhibited by TRIS (*K*_i_ = 12 mM) and most importantly by phlorizin (*K*_i_ = 0.44 mM). In contrast, phlorizin hydrolase activity is unaffected by lactose, confirming the existence of multiple catalytic sites leading to discovery of the phlorizin hydrolase subunit on LPH [11, 60]. The phlorizin-site catalyses the hydrolysis of phlorizin to phloretin and glucose (Fig. 7a). The endogenous substrates have been hypothesized to be glycosylceramides [29, 56]. Both catalytic sites of LPH hydrolyze the substrates as retaining *β*-glycosidases (Fig. 2a) [26, 61]. The critical difference in substrate preference between the substrate sites is the aglycon. The lactase site of the enzyme prefers hydrophilic aglycones, while the phlorizin site prefers mainly hydrophobic aglycones (glycosylceramides and aryl-*β*-glycosides) [60, 62, 63]. As the substrate preference for both catalytic sites differ significantly in aglycon specificity we will discuss the lactase and phlorizin hydrolase substrate specificity in separate sections below.

### LPH activity for lactose derivatives

**Fig. 6:**
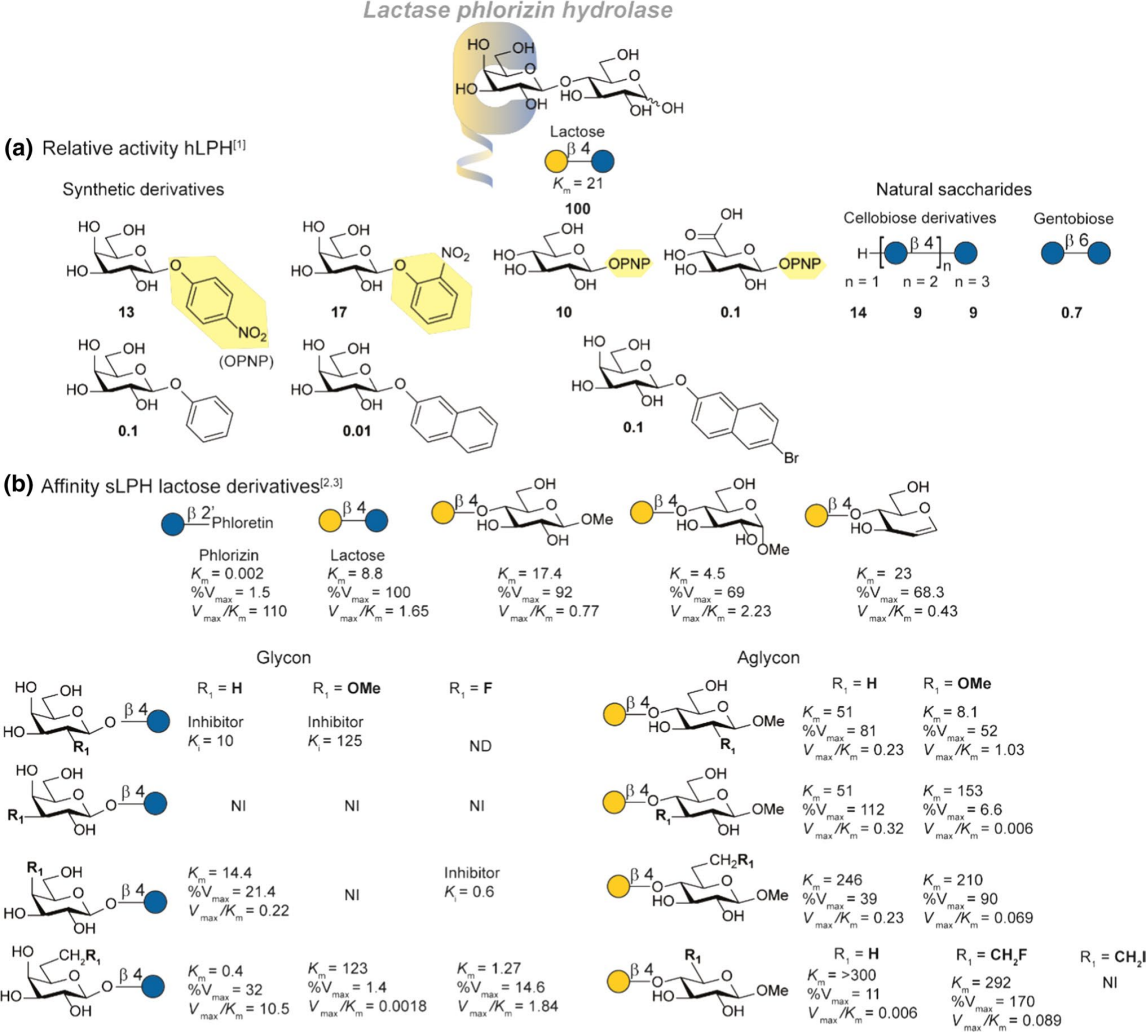
SAR study of the lactase-site of LPH. **a** An overview of the relative activity of hLPH towards various synthetic and natural carbohydrates. The activity is reported in percentages relative to lactose. **b** An overview of the aglycon and glycon affinity of sheep LPH (sLPH) towards synthetic lactose derivatives. *K*_m_ and *K*_i_ are reported in mM, *V*_max_ is reported as percentage relative to lactose. NI (no interaction), ND (not determined), PNP (4-nitrophenol). References used: ref 1 [11], ref 2 [64], ref 3 [59]

Pioneering SAR-studies were performed by Norén et al*.* [11] and Enevoldsen et al*.* [65] with purified LPH from human- and pig intestinal tissue (hLPH and pLPH respectively). The activity for most substrate studied was reported as percentage of the activity of hLPH relative to lactose and is summarized in Fig. 6a. In addition, the *K*_m_ of the natural substrate was measured and compared to gluco-analogue cellobiose (21 mM and 4.4 mM respectively). Synthetic carbohydrate derivatives conjugated to aromatic aglycons showed a decrease in activity compared to lactose. Interestingly, the configuration of the 4-OH of the non-reducing end does not significantly impact the hydrolytic activity (glucose). This is again reflected by the hydrolytic activity towards natural saccharides. Cellobiose, -triose and -tetraose still show moderate activity. Interestingly, LPH can also hydrolyze cellulose although the limited solubility of cellulose diminishes its nutritional value [11]. In contrast, the C-6 oxidized analogue glucuronic acid shows little activity. The limited access to human intestine samples have resulted in most other studies using LPH from other mammalian species such as sheep LPH (sLPH). The substrate specificity of sheep sLPH was determined by Martín-Lomas et al*.* [59, 64] with synthetic lactoside derivatives (Fig. 6b). It is expected that the *K*_m_ and *V*_m_ values obtained in this study are only for the lactase subunit since cellobiose and lactose do not inhibit the phlorizin hydrolase. This is underlined by hydrolysis studies which show that methyl *β*-lactoside as well as lactal are exclusively hydrolyzed at the lactase site [64]. Since the *α*/*β*-equilibrium in case of reducing end sugars complicates specific *K*_m_ determination, the lactoside analogues studied were substituted at the reducing end. Comparison of the methyl *α*-lactoside and methyl *β*-lactoside showed both are hydrolyzed by sLPH, although there is a slight preference for the α-anomer. This is in line with the observation of Norèn et al*.* (cellotriose, cellotetraose and cellulose are hydrolyzed) that C-1 modifications on *n* + 2 position are allowed [64, 65].

Systematic deoxygenation, methylation of fluorination of the hydroxyls of the galacto-moiety reveal an interesting trend in glycon-substrate specificity. Deoxygenation or methylation of the galactosyl C-2′ hydroxyl results in a competitive inhibitor which is most pronounced in the case of the C-2′ deoxy derivative (*K*_i_ = 10 mM). It has been proposed that this HO-2′ is vicinity of a carboxylate group in the active site and is necessary to stabilize the intermediate glycosyl oxocarbenium ion or is involved in the formation of a covalent galactosyl-enzyme intermediate [59, 64, 66]. Modification of the C-3′ position is not permitted in all studied derivatives and showed neither hydrolysis nor inhibition, indicating that the HO-3′ is a critical polar group for substrate recognition. Most likely, the C-3′ position is involved in hydrogen bond donation [59, 66]. The C-4′ position modification are tolerated as cellobiose and other glucosides are hydrolyzed by lactase [64]. Hence, deoxygenation retains affinity though a decrease in hydrolysis rate is observed. Conversely, methylation or fluorination leads to loss of affinity or inhibition respectively. A C-4′ fluor derivative is an inhibitor suggesting that it fits in the active site, but the fluorine likely destabilizes the reaction pathway via the oxocarbenium cation due to its electronegativity [59]. The trend observed with respect to C-4′ modification suggests LPH to be classified as a *β*-glycosidase instead of only a *β*-galactosidase. The C-6′ deoxy derivative showed even higher affinity than methyl *β*-lactoside, indicating that a hydrophobic environment is present in the active site on this position. High affinity was also observed in case of the 6′-deoxy-6′-fluoro lactoside. In contrast to the C-4′ fluor derivative, the C-6′ fluor is hydrolysed, suggesting a different impact on the oxocarbenium cation. Larger substituents at the C-6′ position were poorly hydrolysed.

In extend, the aglycon substrate specificity was studied. It was proposed that the glucose moiety is important for binding since lactulose [65] and *β*-d-galactopyranosyl-(1 → 4)-1,6-anhydroglucose are not hydrolysed by lactase. The C-2-deoxy and C-3- deoxy derivatives of the glucose moiety show only a similar threefold increase in *K*_m_, suggesting that they play a small role in substrate recognition. Methylation at these positions, however shows a dramatic decrease in catalytic efficiency (*V*_max_/*K*_m_) in case of C-3 whereas C-2 methylation is relatively unaffected. These observations indicate that though HO-3 is not critical for binding, the local environment is sterically crowded. The C-5/C-6 positions seem least tolerant to modifications as deoxygenation, methylation, fluorination, iodination or the xylose derivative all show sharp increase of *K*_m_. This indicates that the HO-6 is important for efficient enzyme binding and it probably participates as a hydrogen bond donor in the periphery of the binding site.

These SAR-studies on glycon and aglycon summarize the substrate preference of LPH towards lactose derivatives. To summarize, the C-2 and C-3 position of the glycon are crucial for substrate recognition and catalytic activity. Small structural modifications on the C-4 are allowed to a certain extend whereas the C-6 position shows more tolerance. In all cases, methylation results in loss of activity suggesting no space for extension at the non-reducing end. At the + 1 (aglycon) site modifications are allowed at C-1 and C-2 and to a lesser extend at C-3. The C-6 positions in contrast is crucial for substrate affinity.

#### LPH activity for hydrophobic derivatives

**Fig. 7:**
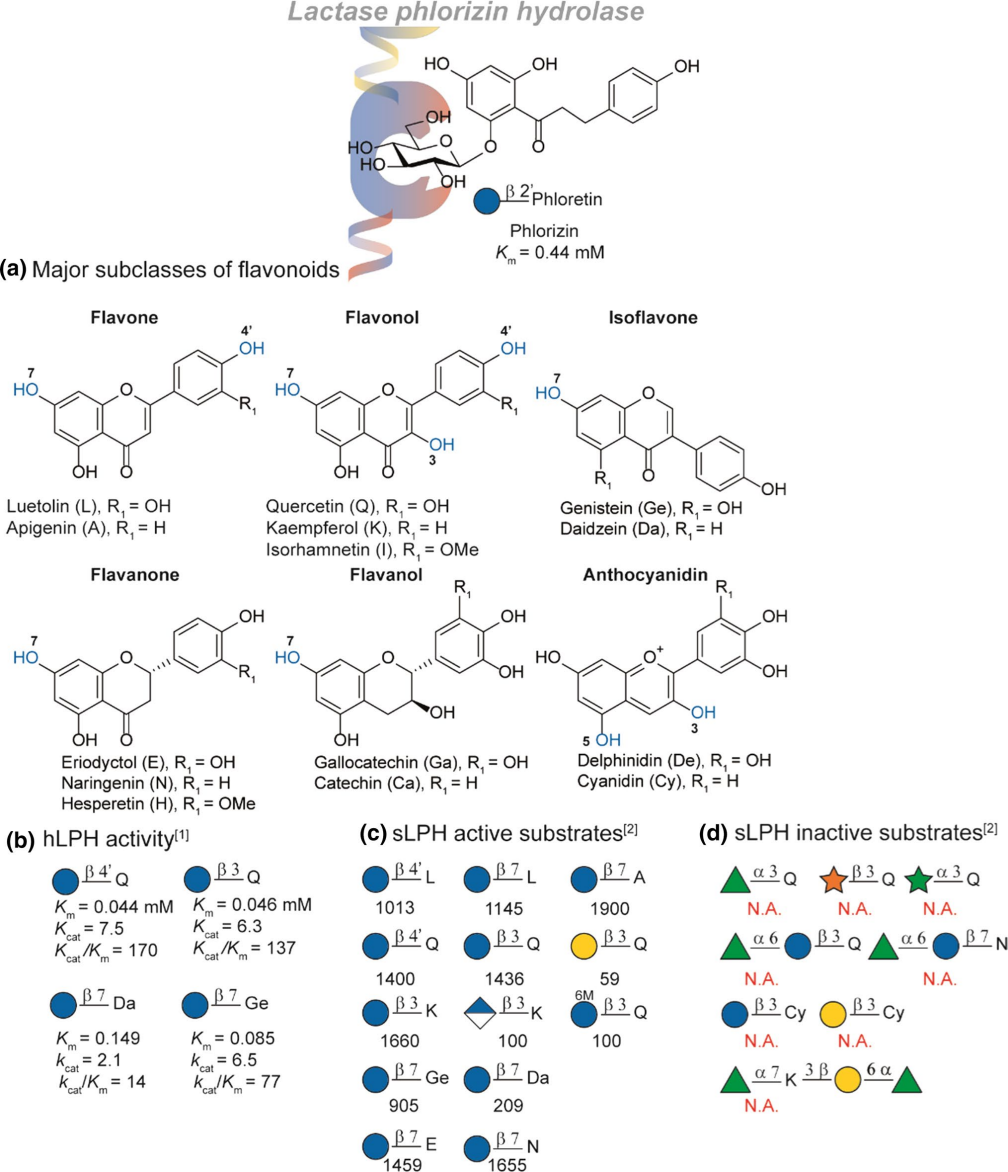
SAR study of the phlorizin-site of LPH. **a** An overview of the flavonoid subclasses. Potential glycosylation sites highlighted in blue. **b** Activity of human LPH (hLPH) towards a selection of flavonoids. **c** Relative activity of sheep LPH (sLPH) relative to phlorizin (100). **d** Overview of the sLPH inactive flavonoids. References used: ref 1 [67], ref 2 [68]

LPH is active against a wide variety of glycosylated flavonoids (for structural overview see Fig. 7a). Though the hydrolytic activity against phlorizin is present at the phlorizin hydrolase site, most flavonoid hydrolysis is observed at the lactase site. This is remarkable since flavonoid derivatives display a high similarity to phlorizin [67]. In the same study, the activity against flavonols (quercetin) and isoflavones (genistein and daidzein) was determined (Fig. 7b). Comparable *K*_m_ and *K*_cat_ was observed in the hydrolysis of the flavanols independent on the substitution site. Interestingly, the meta-substituent relative to the glycosylation site in isoflavones had a high impact showing a five-fold drop in activity in case of non-substituted isoflavone (daidzein-7-glucoside). Additional studies performed with sLPH showed a high activity in most glucosylated flavonoids (Fig. 7c). Similar trends in sLPH activity trend towards daidzein-7-glucoside and the genistein analogue was observed as in hLPH. Though lower compared to the glucosylated analogues, activity was observed towards 3-galactosylated quercetin as was for 3-glucuronated kaempferol. Interestingly, also malonated glucoside-3-quercetin also was hydrolysed by the enzyme. *α*-l-rhamnosylated, *β*-d-xylosylated, *α*-l-arabinosylated or rutinated quercetins proved no substrate as also observed for kaempferol disubstitued with *α*-l-rhamnosylated and rutinose (Fig. 7d). In addition, sLPH did not show activity towards cyanidins. More on the absorption of flavonoids and their absorption model can be found in a paper by Kroon et al*.* [68]. For a comprehensive overview on flavonoid absorption we refer to a review by Jiang et al. [69].

### Cytosylic beta glucosidase

**Fig. 8:**
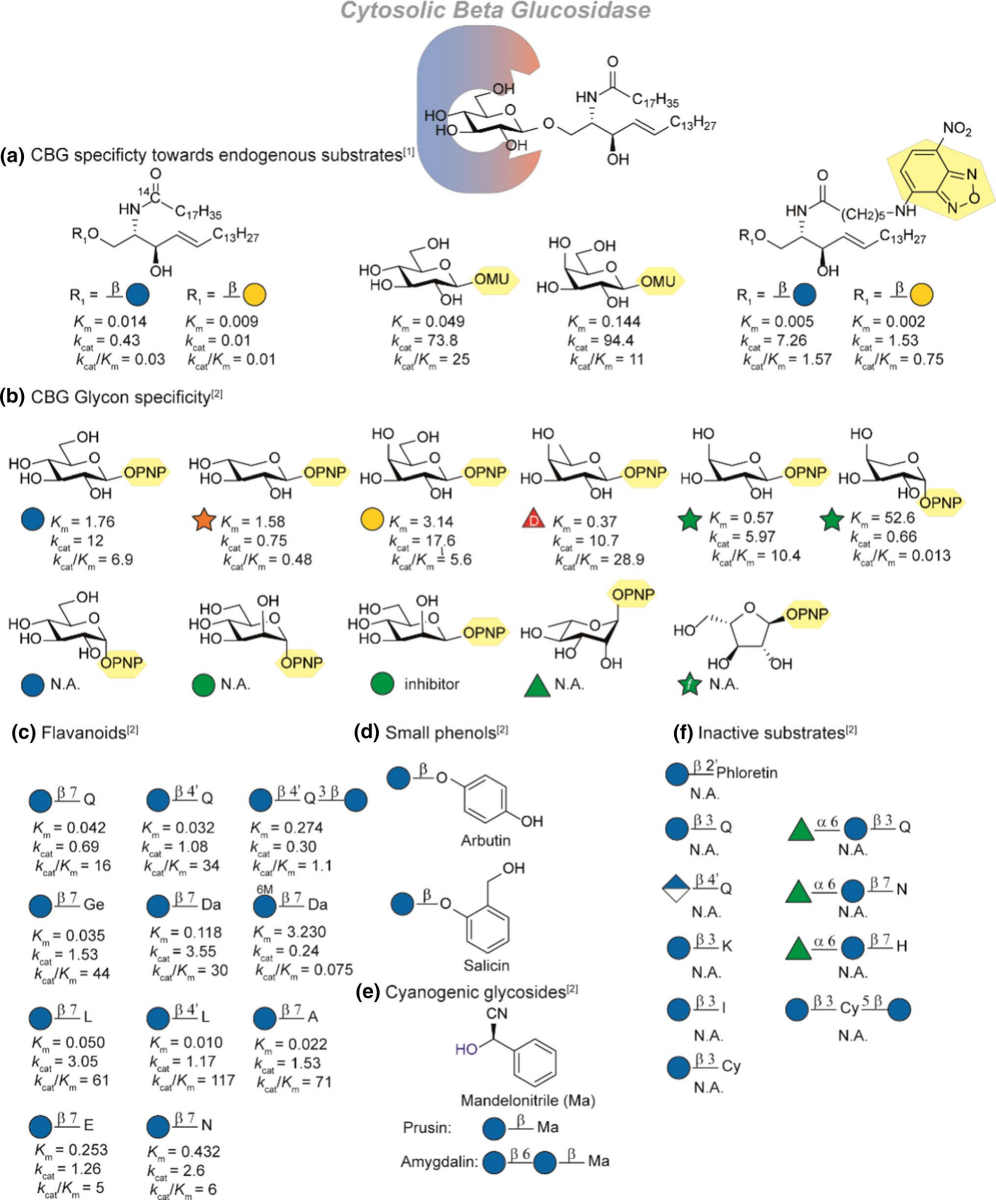
Overview of SAR-studies on CBG specificity: **a** Overview of the activity of CBG towards glycosylceramides (*K*_cat_ in s^−1^). **b** Overview of glycon specificity. Activity towards flavonoids (**c**). Exact activity towards small phenolic substrates (**d**) and cyanogenic glycosides (**e**) were not reported. **f** Studied substrates which were not hydrolyzed by CBG. Referneces used: ref 1 [12], ref 2 [70]. *K*_m_ is reported in mM. N.A. Not Active

Human cytosolic *β*-glucosidase (hCBG, 3.2.1.21) is a broad specificity *β*-glycosidase enzyme involved in the intracellular hydrolysis of *β*-glycosylated xenobiotics and glucosylceramides. hCBG is mainly expressed in the cytosol of the liver, kidney, intestine and spleen [70, 71]. Since hCBG acts as glucosylceramidase (GC) it is also reported as khloto related protein (KlrP). To avoid confusion we will only refer to hCBG [72]. Interestingly, the activity as GC has prompted to study possible involvement of CBG in Gaucher disease, a recessively inherited lysosomal storage disorder resulting. Gaucher disease is normally linked to deficiency in lysosomal GC (3.2.1.45) and cause massive accumulation of glucosylceramide in tissue macrophages leading e.g. enlarged spleen and liver, bone pain and bleeding problems. It was found that in some patients soluble *β*-glucosidase was impaired [73] this observation combined with Hayashi and co-workers’ report about GC activity [12] suggested involvement of CBG. However, findings from the group of Beutler [74] and the group of Aerts [75] disputed this and found hardly any activity towards natural glucosylceramide. In addition, inhibition or impairment of CBG did not show elevated intracellular glucosylceramide levels nor correlation with type 1 Gaucher disease severity. Like all GH1 *β*-glucosidases, hCBG carries two glutamate residues in its active site (Glu^165^ and Glu^373^), that catalyse the hydrolysis with overall retention of configuration in two steps (see Fig. 3b) [76]. First the aglycone is protonated and departs after nucleophilic attack of the glutamate residue resulting in the formation of an *α*-linked covalent glycosyl-enzyme intermediate. The glycone undergoes conformational changes from a chair via a skew boat to the half-chair transition state (^4^C_1_ → ^1^S_3_ → ^4^H_3_), which changes further to the α-linked glycosyl enzyme intermediate in the chair conformation via a skew boat (^4^H_3_ → ^1^S_3_ → ^4^C_1_) [26, 77]. Interestingly, hCBG hydrolytic activity is not limited to glucosides as it can also hydrolyse other hexoses and pentoses (see Fig. 8b). For a full analysis of the crystal structures we refer to studies by Juge and coworkers who employed a model structure together with the crystal structure of hCBG to investigate the aglycone specificity and the structural motifs/domains compared to other GH1 family members [76, 78]. In addition, the crystal structure of the covalent glycosyl intermediate was elucidated by Kakuta et al. [72]. Importantly, they confirmed the double displacement hydrolysis mechanism of hCBG and potential transition state stabilization by a 2-OH water interaction.

Most SAR studies focus on the function of hCBG in the hydrolysis of xenobiotic compounds, the endogenous role of CBG however, was revealed in 2007 as glycosyl ceramidase (GC) [12]. Ito and coworkers confirmed GC activity by incubation of radio- and fluorescent labelled glycosyl ceramides (Fig. 8a). Though a high affinity was found, the overall activity is low compared to simple model substrates such as 4-methylumbelliferyl-(MU)-*β*-glucose and -*β*-galactose. The slow turnover can be explained by the slow rate of aglycon departure which is still tightly bound. In all combinations, glucose showed to be a better substrate than galactose as glycon. Juge and coworkers expressed hCBG from the liver in *Pichia pastoris* (reCBG) and performed a comprehensive study of glycon and aglycon specificity [70]. On the glycon site (Fig. 8b), reCBG showed a preference for equatorial linked sugars as PNP-*α*-glucose is not a substrate for the enzyme [70, 71]. In contrast, a broad variety of sugars was tolerated as long as the C-2 hydroxyl group is equatorially oriented (the epimer PNP-*β*-mannose acts as inhibitor). Remarkably, *β*-d-fucose and *α*-l-arabinopyranose showed a higher *K*_cat_/*K*_m_ value than glucose. Such trends are observed more often in GH1-class hydrolases and the origin has been extensively studied by Withers and coworkers on *β*-glycosidase from *Agrobacterium. *sp. [79, 80].

The aglycon specificity of hCBG has mainly been focussed on xenobiotic *β*-d-glycosides (for structural details on flavonoids see Fig. 7a). A variety of flavonoids was tested with reCBG (Fig. 8c). *β*-d-glucosides of isoflavones, flavonols and flavones were good substrates, while the saturated flavanone glucosides were hydrolysed less efficiently (lower catalytic efficiency constants *k*_cat_/*K*_m_). This indicates that a flat aromatic molecule is a favourable substrate compared to aliphatic analogues. CBG shows a high specificity for regio-chemistry. Glucosylation at the 4′ and 7-position on the flavonoid skeleton are well tolerated whereas in contrast to LPH, substitution at the 3-position leads to loss of catalytic activity. This decrease in affinity of the 3-position by local steric hindrance was later rationalized by modelling studies [70, 76]. Furthermore, malonylation of the glucose moiety e.g. in case of daidzein-7-Glc results in a significant decrease in both hydrolysis rate and affinity. Similar to LPH, *α*-l-rhamnosylation as present in naringenin-7-rutonise and hesperitin-7-rutinose is not accepted. In addition, glucosides of dihydrochalcones (phlorizin), anthocyanins, glucuronides (quercetin-4′-Glu) and were not found to be substrates (Fig. 8f) [70]. The enzyme showed activity towards cyanogenic glucosides (Fig. 8e, amygdalin and prusin) and phenolic glucosides (Fig. 8d, arbutin and salicin) though these were hydrolysed least efficiently (~ 10% and 1% of the average rate observed for flavonoid monoglucosides respectively). Before hCBG is able to hydrolyse these compounds, they need to be transported to the cytosol. Glycoside transporters play an important role in monosaccharide- and glycoconjugate transport over the apical membrane and will be discussed in the next section.

## Glycoside transporters: analysis of affinity and transport

The study of substrate specificity of transporters is challenging compared to enzymes involved in chemical conversion. The latter class can be studied in cell free systems whereas transporters have to be studied expressed in membranes. Standard methods for transport discussed in this review involve radiotracers, electrophysiology or HPLC–MS methods. Radiotracers are e.g. ^3^H-, ^14^C or ^18^F radiolabelled derivatives of sugars which are added to an expression system and their transport can easily be monitored over time. They do however require chemical modifications with radioactive isotopes of the sugar to be studied. Often a standard radio-labelled compound of pre-determined *K*_m_ such as [2,6-^3^H]-2-deoxyglucose is used and the substrates of interest are co-incubated. The decrease in uptake of the radiotracer a *K*_i_-value can be determined but no direct evidence of transport is obtained. Electrophysiology studies are more direct but require an electrogenic transporter such as the sodium dependent glucose transporters (SGLT). *Xenopus laevis* oocytes are often used as expression system of the human-cDNA encoding for the transporter of interest. Next, they are used in combination with two-electrode voltage clamp or patch-clamp techniques. The measures current is dependent on the substrate and its concentration and form a reliable system to study direct substrate affinity (*K*_m_) and even transport (*V*_max_). Alternatively, transport studies involving HPLC–MS analysis can be used in combination with a cell line that can grow into a monolayer system mimicking the intestine such as human colon cancer cell line Caco-2. The substrates of interest can be added at the apical or basolateral side and their transport can be measured with HPLC–MS. Care has to be taken as Caco-2 is a cancer cell line which can also express elevated levels of GLUT1, and in addition their expression into a monolayer is slow and time-consuming. Finally, it goes without saying that specific transport has to be assessed with the correct control experiments, requiring use of a known specific inhibitor as the situation especially in multi-cell systems may not be trivial.

### Apical transport

The major first pass carbohydrate transporters involved in intestinal monosaccharide uptake are the active sodium dependent glucose transporter (SGLT1) and the passive facilitative fructose transporter (GLUT5). Although both transporters differ in uptake mechanism and substrate specificity, the transport is coupled to a phosphorylation pathway that ensures a positive gradient over the membrane (Fig. 1). Interestingly, the involvement of other transporters in monosaccharide uptake like GLUT7 [81], GLUT9 [82] and GLUT12 [83] are suggested in literature but under debate as their exact role in monosaccharide transport awaits to be revealed [84, 85]. Since these transporters are expressed to a much lower extend than SGLT1 and GLUT5, this review will focus on the latter two.

#### Carbohydrate transport by SGLT1

SGLT1 (SLC5-family) is the major glucose-transporter of the intestinal tract [86] and is mainly expressed, but not limited to, the brush border membrane of the small intestine. Additionally, SGLT1 is expressed in the trachea, heart brain and various other tissues (Table 1). First cloned and studied by Wright and coworkers in 1987 [87], SGLT1 selectively transports glucose and galactose with millimolar affinity [13, 88] (*K*_m_ = 0.5 mM for both substrates) over the apical membrane (Fig. 1, top right). Transport of d-glucose is sodium dependent in a stoichiometry of one monosaccharide per two sodium ions and is therefore a symport transporter. This means that glucose absorption is not limited to high apical glucose levels obtained after consumption but also in between meals when a low glucose concentration is present. The sodium gradient is maintained by Na^+^/K^+^-ATPase which provides extracellular transport of three sodium ions for two potassium ions, gaining one charge per cycle. SGLT1 is also capable of facilitating water transport which is unaffected by substrate binding [89]. Defects in the SGLT1 gene are related to the autosomal recessive disease glucose/galactose malabsorption which is characterized by failure of glucose and galactose absorption in the small intestine [90].

#### Transport mechanism of SGLT1

**Fig. 9:**
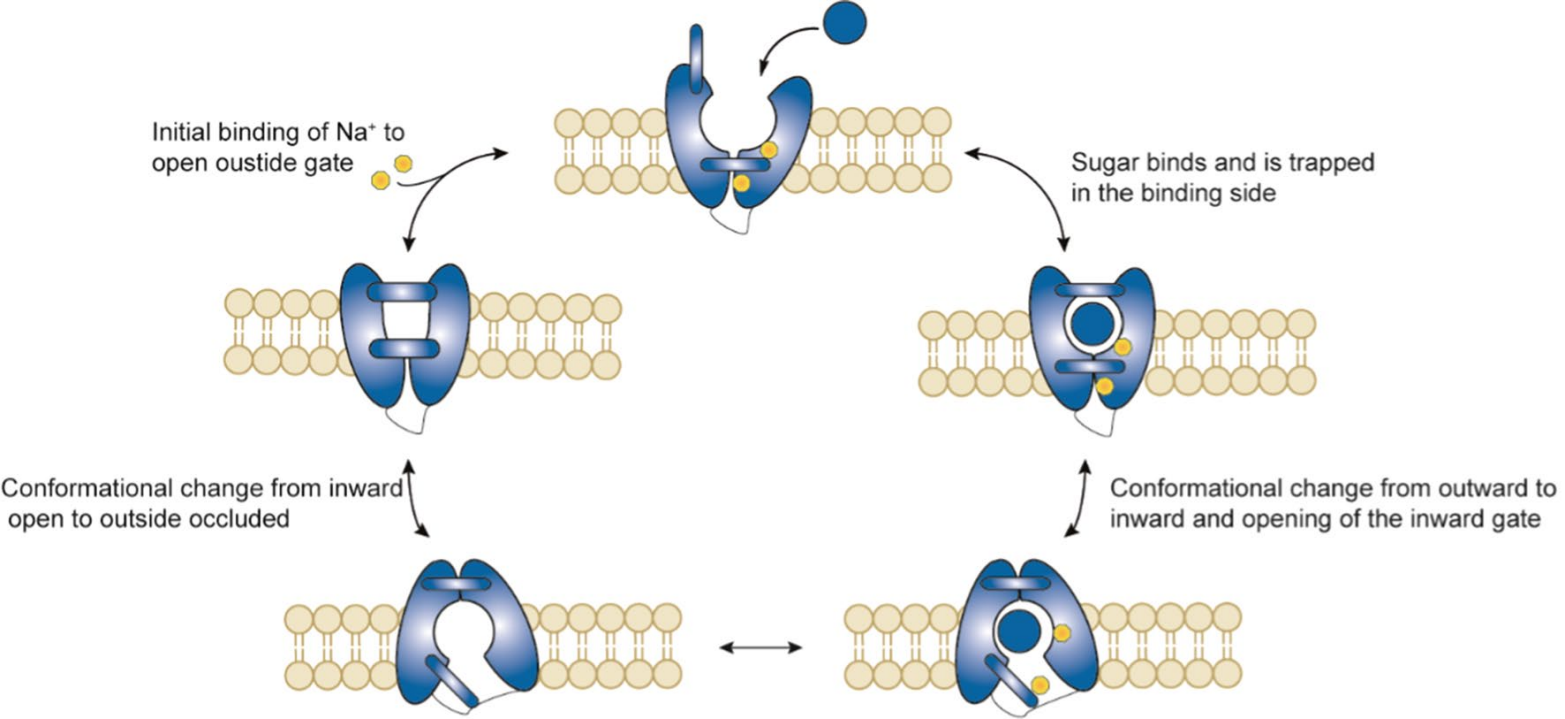
Schematic overview of the proposed 5-state model of SGLT1 during glucose transport ( adapted from Wright and coworkers, 2017) [91]

Although no crystal structures of human SGLTs are known thus far, a crystal structure of a bacterial homolog (vSGLT) [92] with a high similarity has been used as a template to model hSGLT1 [93, 94]. In addition, recent studies by Lapointe and coworkers showed that SGLT1 is expressed as a homodimer via an extracellular disulfide bridge [95]. Figure 9 shows the proposed six state mechanism of hSGLT1. The first step in the process involves the binding of Na^+^ in the binding pocket to open the outside faced gate. Interestingly, binding of the first Na^+^ promotes binding of the second Na^+^ and also sugar binding [96]. In the next step, the sugar binds in the pocket and is trapped by closure of the outside gate. The binding of glucose and closure of the outside gate causes an allosteric interaction and changes the conformation from outward occluded to inward occluded. Subsequently, the inward gate is opened to release glucose and the sodium. It should be noted that the rate of transported is governed by the opening and closing of the outer and inner gates [91]. After dissociation of the substrate, the inward gate closes and the protein rests in the inward facing ligand free state. The final step, before the process can repeat itself, is the conformational change from inward facing to outward facing ligand free state after which sodium can bind in the pocket again to open the outside gate. At close to *V*_max_ conditions, the turnover rate (defined as the number of complete cycles each protein performs per second) was 13.3 s^−1^ based on the uptake of α-methyl glucose. For a detailed study of the structure and kinetics of human SGLT1 we refer to Wright and coworkers [94].

#### Substrate affinity of SGLT-1

**Fig. 10:**
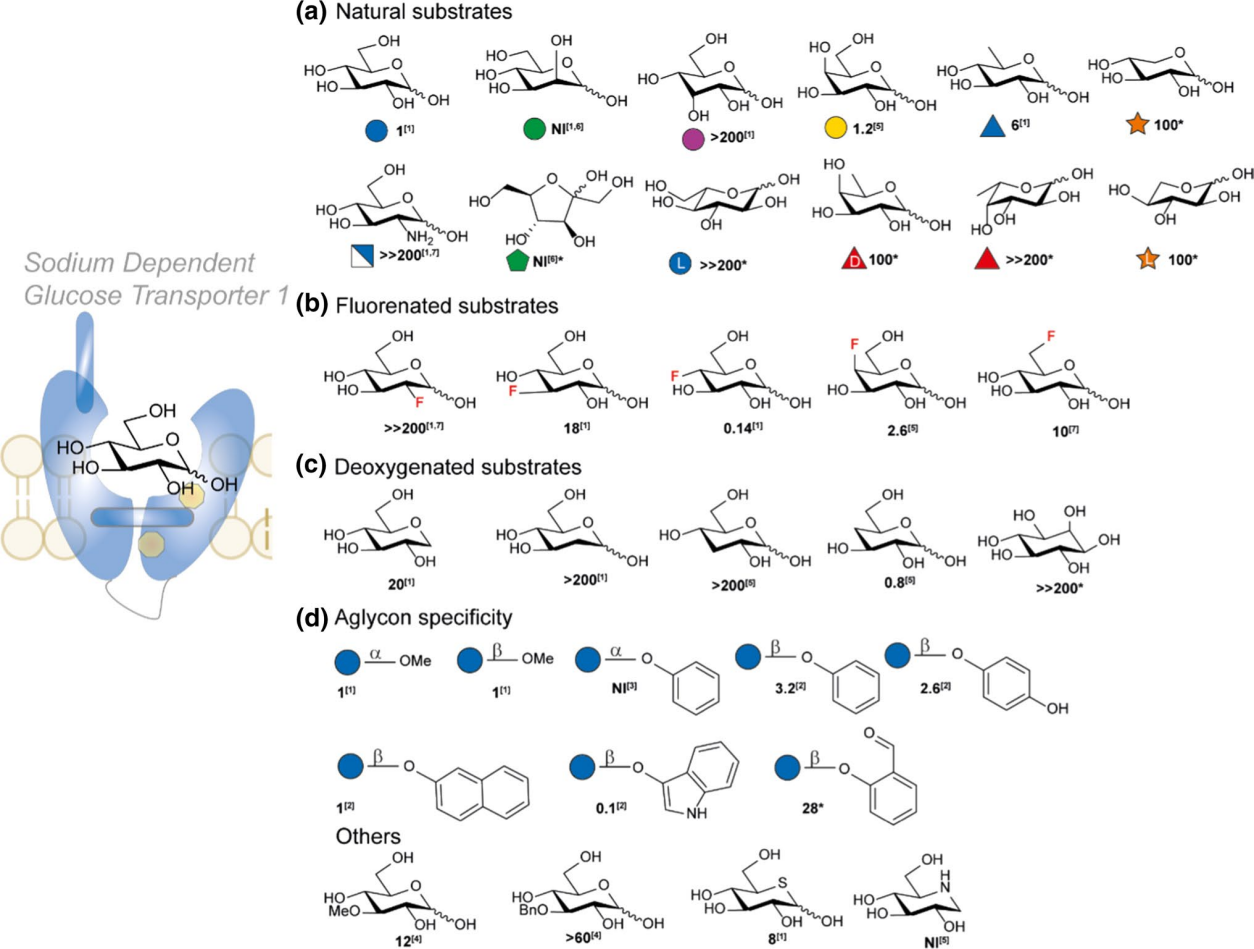
Relative Substrate Affinity of hSGLT1 [93] grouped according to chemical substitution in natural substrates (**a**), fluorinated substrates (**b**), deoxygenated substrates (**c**), glycosylation (**d**) and other substitutions (**e**). *K*_0.5_ values were obtained using a two-electrode voltage clamp method on *Xenopus laevis* oocytes. *I*_max_ in the substrates transported equalled ~ 83% of the *I*_max_ of methyl *α*-glucose. All values reported are relative to the *K*_m_ of methyl *α*-glucose (0.5 mM). References used: ref 1 [13], ref 2 [97], ref 3 [98], ref 4 (or reported herein as unpublished data) [99], ref 5 [100], ref 6 [101], ref 7 (reported as unpublished data) [93]. The O-1 interaction could not be modelled. *K_m_’s of rabbit SGLT1 (rbSGLT1) [102]

Although no crystal structure of human SGLT1 is known yet, SGLT1 is arguably the best studied human monosaccharide transporter. Since the transport of the sugar is directly related to the transport of sodium, two-electrode voltage-clamp techniques can be used to study the substrate transport and affinity. Figure 10 depicts a overview of the substrate affinity of hSGLT1 [93]. The transport of a wide variety of natural monosaccharides and synthetic monosaccharide derivatives have been tested in oocytes and are grouped accordingly. The affinity is reported as *K*_0.5_ at constant *I*_max_ relative to methyl *α*-glucose, details are mentioned in Fig. 10. The trends observed in these studies give crucial information about which carbohydrate substituents determine the affinity of the substrate. d-glucose and d-galactose are the only common natural sugars accepted for transport. Epimers at C-2 (d-mannose) and C-3 (d-allose) lead to complete loss of substrate transport (Fig. 10a). Also the important nutrient d-fructose is not accepted, neither are l-sugars: l-glucose, l-fucose and l-xylose. Interestingly, 6-deoxyglucose (d-quinovose) is still transported though the affinity is six fold lower than d-glucose. Systematic positional fluorination (Fig. 10b) reveals that although the affinity decreases, substitution is accepted at C-3, C-4 (both galacto and gluco-stereochemistry) and C-6. Interestingly, 4-fluorodeoxyglucose (4-FDG) shows a lower *K*_0.5_ than d-glucose. Importantly, fluorination at C-2 leads to complete loss in affinity as is also observed in case of: amino derivatization (2-ADG), and deoxygenation (Fig. 10c). This suggests that substrate-protein interactions are tight at C-2 and there is not much space for chemical adjustments. This might be a consequence of the disturbed hydrogen-bonding with the asparagine and histidine moiety close to the 2-OH and suggests properties of 2-OH as both H-bond donor and acceptor. Deoxygenation at C-4 is tolerated and leads to a slight increase in affinity which suggest that hydrogen bonding is not crucial at this position. In contrast to C-3 fluorination, deoxygenation leads to a dramatic loss of affinity. This is no surprise as previous study have determined C-3 H-bonding with glutamic acid E102 in which O-3 acts as donor. This is further confirmed as methyl, benzyl substitution at C-3 results in a loss up to 60-fold in affinity (Fig. 10e). Furthermore, mechanistic studies by Wright and coworkers have confirmed the involvement of O-3 in CH/*π*-interactions by mutation of “stacking” residue Y290 that causes severe loss of sugar affinity [94]. Critically, O-1 seems to have the most space, and can tolerate various groups such as methyl, phenyl, and naphthyl without a significant loss of affinity. Surprisingly, indican gives a tenfold improvement in affinity though it is transport is only at 14% of the maximal rate of α-methoxy glucose [103]. It is also worthwhile to mention that the transporter has a preference for *β*-substitution as phenyl *α*-d-glucose is not a substrate for the transporter although a small *α*-linked moiety such as a methyl group is accepted. Finally, the endocyclic oxygen showed tolerance for its thio derivative (only a sixfold decrease). However, replacement by other heteroatoms as carbon (myo-inositol) or nitrogen (1-deoxynojirimycin) disturb crucial interactions so a sharp decrease in affinity is observed. Apart from interactions of the hydroxyl-groups, it is hypothesized that additional affinity is obtained by hydrophobic stacking interactions between the axial hydrogens and aromatic residues [13]. The information obtained from these studies are of importance regarding rational design and synthesis in targeting SGLT1 and suggest that chemical modification of C-1, C-4 and C-6 has the most potential.

#### Carbohydrate transport by GLUT5

GLUT5 was first isolated in 1990 by Bell and coworkers [104] and in 1992 confirmed as the major intestinal d-fructose transporter [17]. GLUT5 is also expressed in the kidney, sceletal muscle and adipose tissue (Table 1). GLUT5 is involved in the passive transport and, unlike SGLT1 [105], is pH and sodium independent [18]. The natural substrate d-fructose has a reported *K*_m_ of 6 [17]–15 mM [18]. Recent studies have shown that the top 10% of the population is now consuming about 75 g of fructose per day which, in combination with the fructose released from sucrose by SI, requires high expression of GLUT5 [106]. The expression of GLUT5 is substrate dependent and is regulated on a transcriptional level [107]. Low expression of GLUT5, as is present in infants and young children, shows a higher chance of fructose malabsorption [108]. However, intestinal fructose malabsorption in adults was not associated with mRNA or protein levels of GLUT5 and the underlying cause is still unknown [109]. To maintain a gradient after consumption, d-fructose is rapidly converted to fructose-1-phosphate by ketohexokinase which is expressed in the enterocytes of the ileum [110]. Determining the *K*_m_ value of the transporter is challenging, since oocyte voltage-clamp measurements cannot be performed. To study the transport, researcher have to rely on radio-isotope labelling, or more laborious methods. This is why most SAR-studies report a *K*_i_ instead. In addition to challenging measurements, d-fructose is present under physiological conditions as both furanose (30%) and pyranose (70%) which may complicate exact affinity determination further. Interestingly, d-fructose seems to bind GLUT5 mainly in the furanose form [18] which suggest a close cooperation between sucrase isomaltase and GLUT5. As the literature reports have been limited a substrate overview has been given mostly based on inhibitory constants (Fig. 12).

#### Transport mechanism of GLUT5

**Fig. 11:**
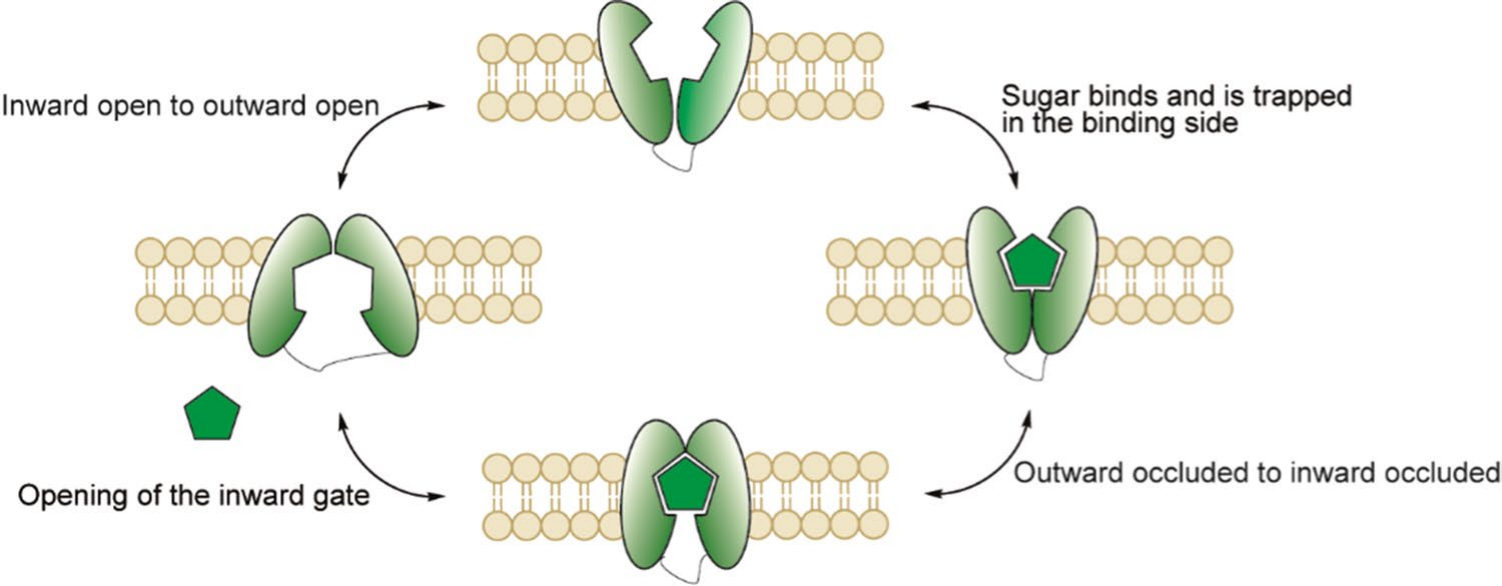
Proposed mechanism of GLUT5 based on the model of facilitative membrane transporters

The exact mechanism of GLUT5 is still under investigation. The proposed mechanism consists of four major conformations comprising two open- and two bound states (Fig. 11). First, the outward open conformation binds the substrate and forms the outward facing occluded state. Next, the outward facing occluded state changes to the inward facing occluded state. Finally, d-fructose releases and the GLUT5 finishes the cycle in the inward open conformation. Returning to the outward open conformation is speculated to be the rate limiting step in the general mechanism of these types of transporters [111]. Crystal structures of both the inward- and outward occluded states of GLUT5 derived from mammalian species *Rattus norvegicus* and *Bos Taurus* were reported in 2015 by Nomura et al. [112]. However, the details about the transitions between the four states are still under investigation.

#### Substrate affinity of GLUT5 based on inhibition

**Fig. 12:**
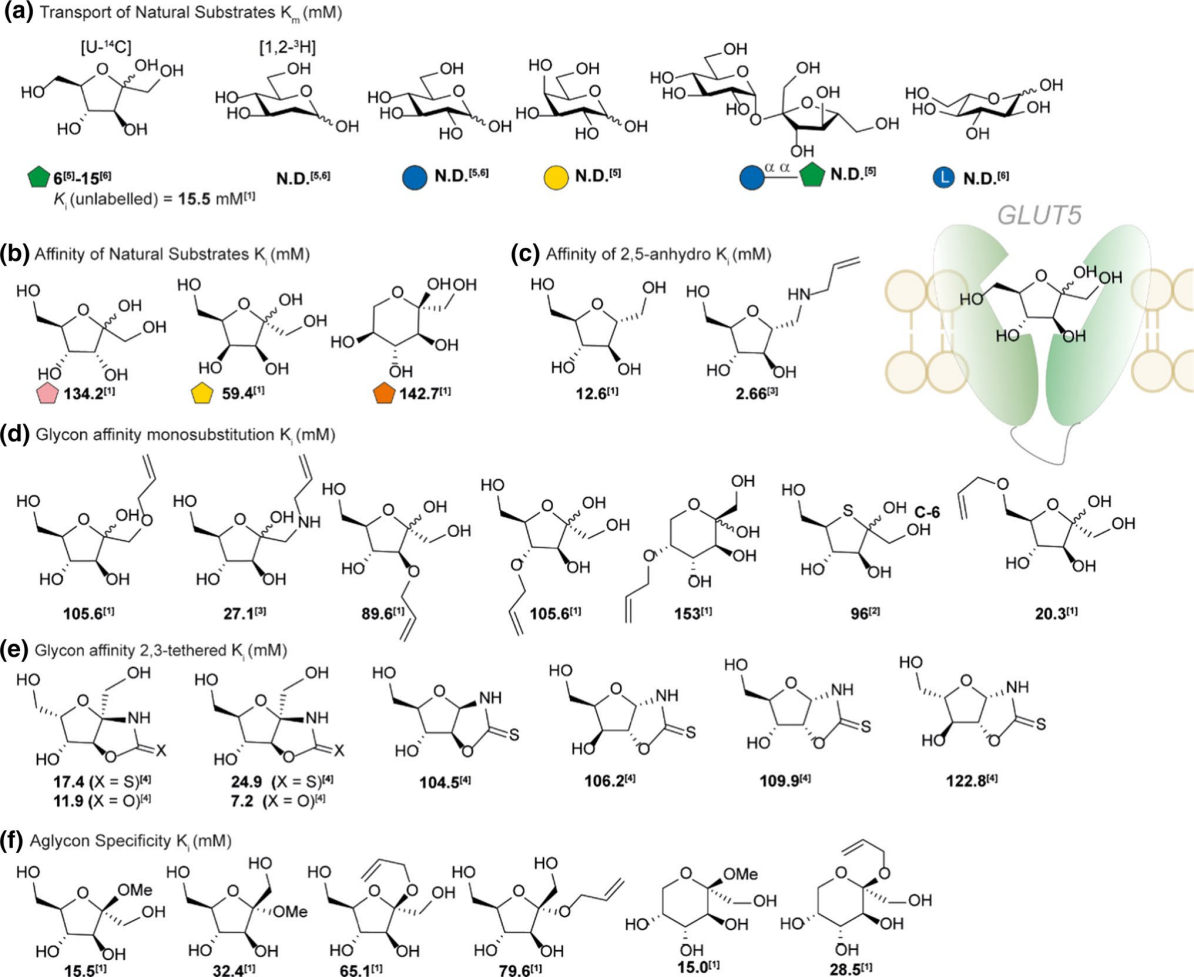
SAR GLUT5. **a** Substrates *K*_i_ was determined relative to the inhibition of a radioactive tracer [^14^C-U]-d-fructose [113]. References used: ref 1 [113], ref 2 [114], ref 3 [115], ref 4 [116], ref 5 [17], ref 6 [18]. N. D. not detected

The most comprehensive studies about the substrate specificity of GLUT5 were performed by Holman and co-workers in the early 2000′s. The method to determine selectivity is based on a CHO-cell line in which rat GLUT5 is expressed at high levels [117]. The *K*_i_ of the substrates are determined relative to the inhibition of the radioactive tracer [^14^C-U]-d-fructose [113]. Substrates tested range from epimers of d-fructose, mannitol derivatives [113, 115] to chemically modified d-fructose analogues at position C-1 to C-6 by alkylation [113], amination [115], C-5 thiolation [114] or C-2/C-3 tethering [116] (see Fig. 12b–e). Natural epimers of d-fructose at C-3 (d-psicose), C-4 (d-tagatose) and C-5 (l-sorbose) result in loss of affinity, most significant in case of l-sorbose which is mainly present in pyranose form. Binding and interaction of d-fructose with GLUT5 was studied by positional allylation (Fig. 12d). Allylation of C-1 leads to a sharp decrease in affinity, whereas amino-allylation is better tolerated, suggesting there is a hydrogen bond donor interaction. This is also suggested from the trend observed in the locked C-2, C-3 oxazolidinone and oxazolidithione derivatives in which all aldopentoses show a sharp loss in affinity (Fig. 12e). Allylation at the C-3 position and C-4 is unfavourable and leads to a loss in affinity which was already observed in case of epimers d-psicose and d-tagatose. Interestingly, FDA-approval of d-psicose (also referred to as d-allulose) in 2014 as safe by the FDA has sparked interest of the community as replacement of sucrose in the diet for obese and diabetic subjects. Critically, recent studies have showed that the substrate can be transported by GLUT5 [118, 119]. Alkylation of C-5 gives the sharpest decrease in affinity, which may be the result of increasing ring size and limited chemical space. In addition, 5-OH substitution by a thiol also leads to a decrease in affinity suggesting hydrogen bond accepting interactions of the endocyclic oxygen with GLUT5. Interestingly, in contrast to C-1 to C-5, allylation of the C-6 position is well tolerated suggesting there is some chemical space. This is also observed in the furanose locked C-2, C-3 oxazolidinone and oxazolidithione derivatives as both d-fructose as l-sorbose derivatives show good affinity.

Finally, substitution at C-2 (aglycon derivatives) shows that small substituents such as a methyl group are tolerated whereas allylation leads to a decrease in affinity. Surprisingly, the pyranose form is well tolerated. Again we note that all derivatives with an open anomeric centre can be present as furanose/pyranose mixture which makes it hard to determine the preferred substrate. A special case is C-2 deoxygenated analogue of d-fructose: 2,5-anhydro-d-mannitol (2,5-AM, Fig. 12c). The symmetrical sugar 2,5-AM has a good affinity for GLUT5 and is only present in furanose form. Critically, substitution at C-1 results in increase in affinity. The decrease in complexity and potential conjugation site at C-1 has been the main inspiration of 2,5-AM for GLUT5 specific probes and will be discussed below.

#### GLUT5 affinity based on molecular probes

**Fig. 13:**
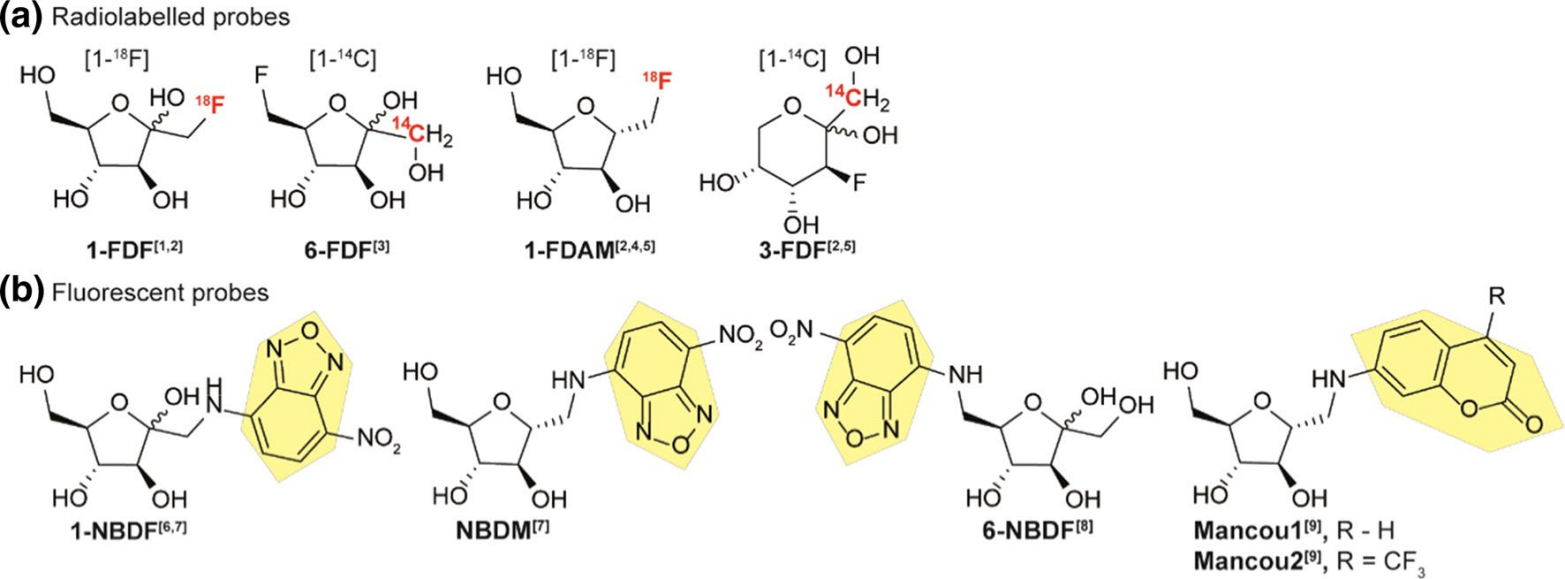
Radiolabelled (**a**) and fluorescent probes (**b**) used in GLUT5 mediated uptake studies. References used: ref 1 [120], ref 2 [121], ref 3 [122], ref 4 [123], ref 5 [124], ref 6 [125], ref 7 [126], ref 8 [127], ref 9 [128]

Most GLUT5 transport studies utilized either radiolabelled (^14^C, ^18^F)- or fluorescent probes (Fig. 13). First studies by Davidson in 1992 confirmed the transport of radiolabelled [^14^C]-d-fructose by GLUT5 [17]. From a different perspective, Masuda and coworkers synthesized and evaluated 1-[^18^F]-deoxy-d-fructose (1-[^18^F]-FDF) as a metabolic analogue of 2-[^18^F]-FDG [120]. Though they noticed initial high uptake in GLUT5 expressing organs kidney liver and small intestine, the probe had no features of metabolic trapping by phosphorylation and was discarded as suitable PET-tracer. Inspired by the tolerance for C-6 modification in Holman studies, Trayner et al*.* synthesized non-radiolabeled 6-deoxy-6-fluoro-d-fructose (6-FDF) as potential PET-tracer in breast cancer tissue [122]. 6-FDF showed inhibition of d-fructose transport mediated by GLUT2 and GLUT5. More important, the radiolabelled variant [1-^14^C]-6-FDF showed accumulation in breast cancer cells with a two-fold increase over [U-^14^C]-d-fructose. Transport of [1-^14^C]-6-FDF was not affected by GLUT2 inhibitor cytochalasin B suggesting the majority of uptake taking place via GLUT5. A follow-up study by Cheeseman and coworkers with 6-[^18^F]-FDF again showed C-1 phosphorylation by recombinant KHK which supports metabolic trapping of 6-FDF [129]. In contrast, incubation with recombinant human hexokinase-II did not result in phosphorylation since the 6 position is blocked by fluorination. Interestingly, the uptake of 6-[^18^F]-FDF was highly dependent on the cell line as a much lower accumulation was observed in MCF-7 cells than EMT-6. Comparison with 2-[^18^F]-FDG showed no improvement on the latter. Next, also inspired by the trends Holman observed, Niu et al*.* explored radiolabelling of 2,5-anhydro-d-mannitol with the synthesis of 1-[^18^F-]-anhydro-d-mannitol (1-[^18^F-]-DAM) [123]. 2,5-anhydro-d-mannitol has the advantage of being symmetrical and eliminates the possibility for furanose to pyranose interconversion thereby reducing the complexity of substrate studies. The compound was evaluated in a MCF-7 rabbit model which showed only a slight increase compared to normal breast tissue. High accumulation was observed in the liver (fructokinase-rich), kidney and bladder conform with the excretion pathway of 2,5-AM. 1-[^18^F-]-DAM was also used in a study by Soueidan et al*.* in addition to 3-deoxy-3-fluoro-d-fructose (3-[1-^14^C]-FDF) with the latter existed mainly in pyranose form [124]. Both analogues were readily taken up in MCF-7 breast cancer cell lines with low IC_50_ values compared of 3.96 and 2.37 µM, respectively against 100 µM [^14^C]-d-fructose. In a recent study by Wuest and coworkers, fructose PET radiotracers 1-FDF, 6-FDF and 1-FDAM were compared with 2-FDG and 6-FDG in murine BC model EMT-6 [130]. In the data presented all d-fructose derivatives were linked with GLUT5 protein levels in tissue. 6-FDF was found to give the highest tumor *vs* muscle contrast among the fructose derivatives, however all showed washout of the tumor due to absence of metabolic trapping by hexokinase II. Involvement of GLUT2 was speculated as EMT-6 have a low GLUT5 expression, marking 6-FDF as a substrate for both transporters. Though the application of d-fructose as PET-radiotracer might be not as suited as 2-FDG, the trends observed clearly show that C-6 fluorinated substrates are accepted as GLUT5 substrates and are less prone to metabolic trapping.

Holman and coworkers reported the first GLUT5 specific photo labelled fructose substrate in 2002 which was introduced at the C-6 position of 2,5-anhydro-d-mannitol. Their probe was reported to show a tenfold affinity over d-fructose [115]. In contrast to the mannitol derivatives, Gambhir and coworkers synthesized two fructose derivatives to Cy5.5 and NBD via the C-1 amine (NBDF). The uptake was assessed in MCF7 breast cancer cells with both probes showing good uptake. Interestingly, close comparison to cells lacking the GLUT5 still showed uptake of the Cy5.5 conjugate, suggesting GLUT5 independent uptake. The NBD dye did show GLUT5 dependent uptake underlining the possible interference of large fluorophores on the substrate recognition of smaller molecules [125, 131]. It must be noted that this result is still under debate as NBDF is sensitive to both d-glucose and d-fructose in uptake studies [125] and was also found to be inhibited by d-glucosamine [126], a substrate for GLUT2. Tanasova et al*.* continued on 2,5-AM derived fluorescent probes and synthesized NBD derived 1-amino-2,5-AM conjugate (NBDM) and evaluated the probe in MCF-7 cells, NBDF and NBDG were added for comparison [126]. NBDM was transported twice as efficiently compared to fructose GLUT5. In addition, fructose preconditioning (fructose rich-medium) resulted in twice as much NBDM transport. NBDM transport was not inhibited by d-glucose and d-glucosamine, pointing towards GLUT5 mediated transport. An interesting SAR was performed by Soueidan et al. based on the synthesized C-6 NBD conjugates of both d-fructose (6-NBDF) and the C-3 (6-NBDP), C-4 (NBDT) and the C-5 (6-NBDS) epimers [127]. 6-NBDF showed rapid take up into MCF-7 and EMT-6 cell lines. Surprisingly, epimers 6-NBDP, 6-NBDT and 6-NBDS showed uptake via GLUT1 instead. This confirms again the hypothesis of Holman that modifications at C-3, C-4 and C-5 are poorly tolerated. In a follow-up study, this strategy was also applied to 2,5-AM and the influence of modification at the C-3 position on d-glucose and d-fructose uptake in EMT-6 cells was studied [121]. H-bond donation was found as requirement as C-3 for interaction with GLUT5. A C-3 NBD derivative of fructose was evaluated for transport in cells and major transport was found via GLUT5 although a minor drop in uptake in presence of Cytochalasin B (GLUT1 inhibitor) could not exclude transport by GLUT1. A *K*_m_ in *Xenopus laevis* oocytes was found to be 1.2 mM, significantly lower than d-fructose. This suggest great promise for application of C-3 derived fructose conjugates in cellular uptake by GLUT5 as long as a spacer is taken with hydrogen bond donating capabilities. Finally, a new class of fluorescent probes based on C-6 coumarin substituted 2,5-AM conjugates was evaluated as application for the analysis of fructose uptake [132]. Electron withdrawing groups at the coumarin appeared to positively influence binding strength as uptake rates. Conjugate ManCou1 was reported to have a 156-fold higher affinity than d-fructose. Two of the conjugates were also tested for their potential as for rapid detection in breast cancer models [128]. A significant difference was found in the cancerous cells MCF-7 and MCF10AneoT compared to normal breast cells MCF10A underlining their potential for rapid on-site high-throughput diagnostics.

### Basolateral transport of carbohydrates

After entering the enterocyte via the apical membrane, the nutritional sugars have to translocate to and cross the basolateral membrane to enter the bloodstream. The classical model describing intestinal sugar transport depicts GLUT2 as the main trans basolateral pathway that facilitates basolateral carbohydrate transport. Interestingly, the exact role of GLUT2 as sole pathway has been under debate [133]. GLUT2 null mice showed normal transport rates of glucose appearance after oral administration despite lacking GLUT2 expression in the small intestine [134]. In addition, studies using an isolated intestinal perfusion model without GLUT2 observed normal glucose transport rates, even under accelerated apical uptake [135]. A different pathway was proposed involving glucose-6-phosphate translocase, a protein transporting glucose-6-phosphate to the lumen of the endoplasmic reticulum. Inhibition of glucose-6-phosphate translocase by S4048 showed decreased glucose release in GLUT2^−/−^ mice but not in the wild type. Interestingly, 3-*O*-methyl glucose, a substrate for both SGLT1 and GLUT2 which is not phosphorylated, was not transported in GLUT2^−/−^. These findings suggest that an alternative pathway based on the accumulation of glucose in the endoplasmic reticulum and subsequent membrane trafficking.

### Carbohydrate transport by GLUT2

**Fig. 14:**
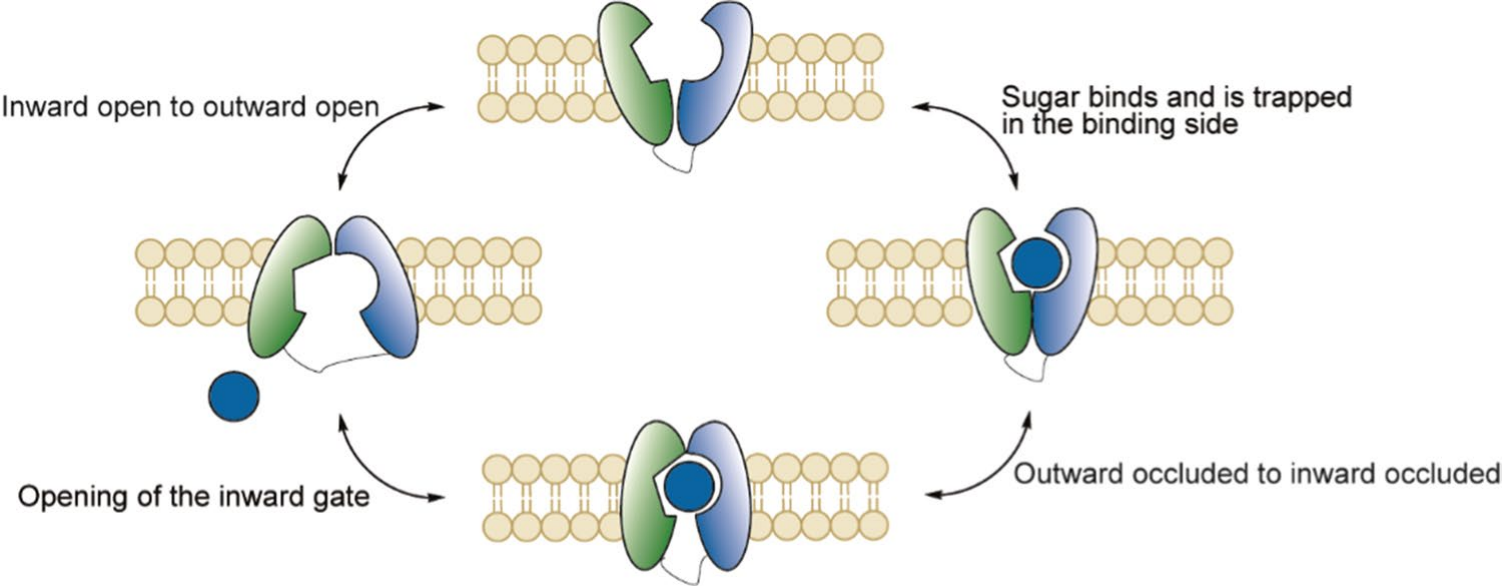
Proposed mechanism of GLUT2 based on the model of facilitative membrane transporters

GLUT2 (SLCA2) is a facilitative glucose transporter, mainly expressed in the liver, intestine, kidney and pancreatic beta-cells [136, 137]. Its function involves: glucose- and fructose uptake [138] and release in hepatocytes, glucose and galactose transport over the basolateral membrane in intestinal cells. Furthermore, GLUT2 plays a critical role in the reabsorption of glucose on the basolateral membrane of the kidney. Defects in the GLUT2 gene cause the Fanconi-Bickel syndrome, leading to pathogenic processes such as hepatomegaly and growth retardation [139]. The affinity for glucose is much lower than SGLT1 (17–20 mM compared to 0.5 mM) but is compensated by a high transport capacity [140]. GLUT2 is presumed to act via the general mechanism based on the model of facilitative transporters (Fig. 14). Critically, the role of GLUT2 in the small intestines has been under debate after studies showed translocation of GLUT2 from the apical membrane to the apical membrane at high glucose concentrations [141, 142]. This may suggest a role in d-glucose uptake under certain conditions and would make GLUT2 a potential target transporter for uptake of exogeneous conjugates. Studies in GLUT2 knock out mice [134] and an isolated intestine perfusion system [135] showed normal glucose uptake indicating GLUT2 as not essential for uptake in the small intestine. A comprehensive study using both SGLT1 and GLUT2 knockouts unequivocally identified SGLT1 as the prime glucose transporter and did not find any evidence for GLUT2 playing a role in glucose uptake at the apical side [86]. This debate was not limited to GLUT2 mediated glucose uptake but also for d-fructose uptake as GLUT5 surrogate [143]. Wild mice fed on a high fructose diet showed a fivefold increase of fructose uptake and only twofold in GLUT2^−/−^ mice suggesting a significant role of GLUT2 in high fructose diets. In contrast, mice with GLUT5^−/−^ showed malabsorption and PKC β-II-mediated GLUT2 insertion did not lead to rescue. Additionally, apical perfusion with high-fructose solutions in neonatal rats increased solely GLUT5 expression [144, 145]. As the exact nature of GLUT2 in the apical membrane remains unclear we will only focus on the better defined role of GLUT2 in basolateral transport.

### GLUT2 substrate affinity

**Fig. 15:**
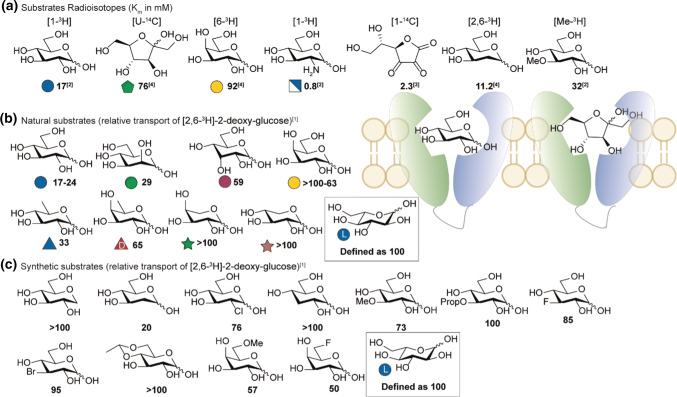
SAR GLUT2. **a** K_m_ of GLUT2 substrates based on radioisotope-labelling. **b** Resulting relative transport of [2,6-^3^H]-2-deoxyglucose (100 µM) against 10 mM of the competing sugar measured in oocytes [16]. Rates are expressed as the percentage of transport of control compound 10 mM l-glucose (set as 100). **c** C-6 derivatives where tested by incubation of 50 mM substrate. References used: ref. 1 [16], ref. 2 [14], ref 3 [146], ref 4 [15]

**Fig. 16:**
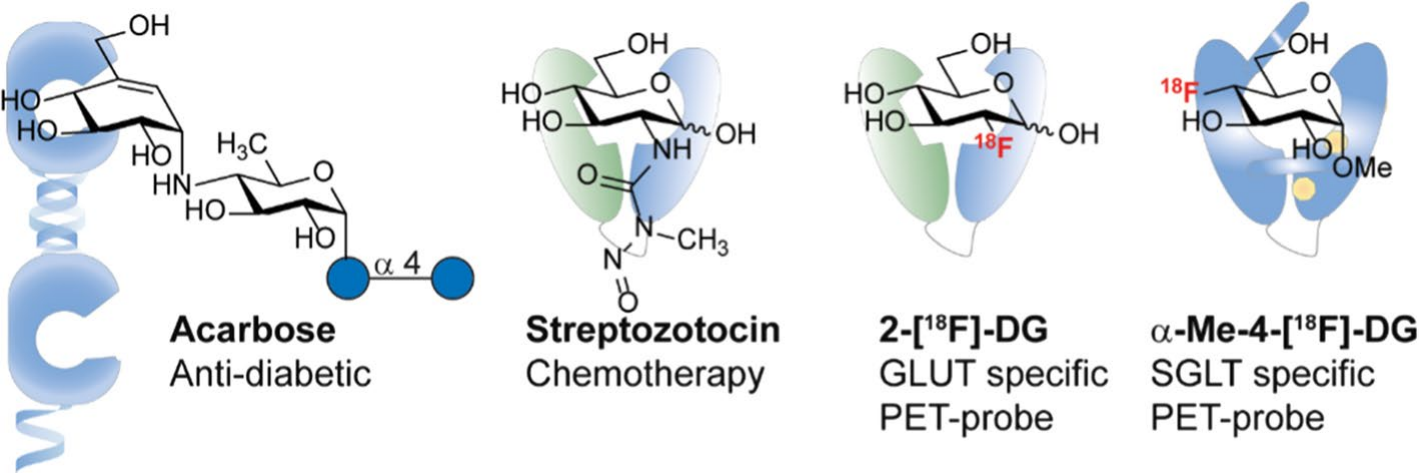
Glycoside derivatives exploited for diagnostics and therapeutics

Similar to GLUT5, no crystal structure of GLUT2 is available. SAR studies in the early 90′s by Gould and co-workers revealed an interesting trend in GLUT2 substrate preference. In a comparative study between GLUT2, -3 and -4, various carbohydrate substrates were co-administered in oocytes and their influence on the transport of radio-labelled [2,6-^3^H]-2-deoxyglucose (*K*_m_ = 11.2) was studied (Fig. 15b, c). l-Glucose did not inhibit uptake of the radioactive tracer and the uptake observed was used as reference (defined as 100) [16]. d-glucose showed a high inhibition of transport, additional studies using a radioactive labelling of glucose later showed a *K*_m_ of ~ 17 mM [14] which is relatively high compared to other GLUT transporters. Addition of 1-deoxy-d-glucose did not alter the uptake of the radioactive tracer. Surprisingly, C-2 derivatives 2-DG and d-mannose show high affinity for GLUT2 whereas chlorination is less well tolerated. 2-[^34m^Cl]-2-deoxy-d-glucose was later evaluated in an animal PET-study and did not show accumulation in GLUT2 rich tissue, instead rapid excretion was observed [147]. The high affinity observed at C-2 was later confirmed in transport studies which showed an exceptionally high affinity of GLUT2 for d-glucosamine (Fig. 15a, *K*_m_ = 0.8 mM) [14]. The *V*_max_ reported (3610 pmol/oocyte/h) was only three-fold lower compared to d-glucose (12,000 pmol/oocyte/h). The sharp decrease of inhibition observed with deoxygenation at the C-3 position suggest involvement in hydrogen bonding. Methylation is tolerated which is also reflected by a *K*_m_ of 32 mM observed in radiolabelled 3-OMG though alkylation with longer alkyl groups is not tolerated. Interestingly, d-allose is a relatively good inhibitor which is in contrast to related transporters GLUT1,-3 and -4. Substitution of C-4 shows no sign of improved recognition by the transporter, this is in sharp contrast to SGLT1 which has a tolerance for C-4. Hence transport of d-galactose by GLUT2 has a high *K*_m_. Interestingly, C-6 derivatives still show inhibition with 6-deoxy sugar d-quinovose as the best inhibitor. Though no transport studies have been performed, this suggest that C-6 derivatives are an interesting target for GLUT2 specific probes. As previously mentioned, GLUT2 is also able to transport d-fructose with a relative affinity of 76 mM. The unique ability to transport C-2 derivatives of d-glucose is most dramatically reflected by streptozotocin toxicity in the islet *β*-cells [148]. This effect has been succesfully employed in combination with chemotherapy in the treatment of metastatic pancreatic neuroendocrine tumors [149, 150]. Other compounds as fructosazine (evaluated in Caco-2) [151] and glycosylated anthocyanins (evaluated in Caco-2) [152] and small glucosamine-conjugated glycopeptides [153] also showed characteristics of GLUT2 mediated uptake though no GLUT2 specific kinetic experiments have been performed. A 6-O-conjugated azomycin derivative did show a GLUT2 dependent toxicity [154]. In contrast to SGLT1 and GLUT5, GLUT2 thusfar has no specific substrate for PET-studies though 2-[^18^F]-DG is proposed to be transporter by GLUT transporters including GLUT2 [155] (see Fig. 16).

## Relevance and conclusion

The wide variety of carbohydrate digestive processes involved in the GI-tract require a broad knowledge for scientists to exploit for diagnostics and therapeutics. To highlight some examples, the GI-tract can already be targeted to influence carbohydrate homeostasis for instance by acarbose (Fig. 16). Acarbose is an antidiabetic drug used to treat diabetes mellitus type 2 by inhibiting α-glycosidase activity in MGAM and SI. This blocks the enzymatic hydrolysis of starch oligo- and di-saccharides to monosaccharides thus reducing blood sugar level. In contrast, transport proteins like SGLT1 are unaffected so monosaccharide transport can still take place. In addition, patients can eat controlled amounts of pure glucose to prevent hypoglycaemia.

In the field of diagnostics small differences in preferred affinity can be used to discriminate between SGLTs and GLUTs. Transporters like SGLT1 allow C-4 modification of substrates whereas C-2 modification leads to loss in affinity. The opposite is applicable for GLUT transporters like GLUT2. This is most clearly demonstrated by the use of 2-[^18^F]-DG as GLUT specific PET-tracer [155, 156] and α-methoxy-4-[^18^F]-d-glucose SGLT-selective PET-tracer [98] and allows the latter to study SGLT-rich regions such as the kidney, skeletal muscle, heart and liver [93]. Importantly, glycosides of drugs may be useful to improve intestinal absorption, targeting to the colon, reduction of gastrointestinal disturbances and to improve solubility.

### Improved intestinal absorption

It has been extensively reported that absorption of quercetin from its glucosides is improved compared to absorption of quercetin itself (see Fig. 7a for structures). The intact glucoside conjugates were not observed on the serosal side of the intestinal membrane, suggesting that hydrolysis is performed on the outside of the enterocytes by LPH or on the inside by CBG. Subsequent studies revealed that the hydrolysis rate of quercetin-4′-glucoside is 10 times higher than the corresponding quercetin-3-glucoside [157]. It was found that SGLT1 plays a role in the hydrolysis kinetics of the 4′-glucoside as inhibition of the transporter by phloridzin strongly reduces the hydrolysis rate. In contrast, hydrolysis of the 3-glucoside was not affected by phlorizin. Evidently the mechanism of absorption of quercitin from its quercetin-4′-glucoside involves both an interaction with SGLT1 and luminal hydrolysis by LPH, whereas quercetin from its quercetin-3-glucoside appears to be absorbed only following hydrolysis by LPH. Taken together, the results suggest that hydrolysis of the glucosides is required before absorption of quercetin can occur. Apparently, this process brings quercetin in close proximity to the intestinal membrane which facilitates its uptake. In is unclear whether this mechanism holds true for other drug glycosides. Very few results have been disclosed on the improvement of oral bioavailability by glycoside prodrugs of medication. The use of prednisolone-21-*O*-*β*-d-glucoside was reported to produce a two-fold increase of oral bioavailability with respect to prednisolone [158]. Another study disclosed the improved bioavailability of glycuronamide and glycoside prodrugs of fluoxetine [159]. Hydrophilic transportable *N*-linked glycosyl dopaminergic compounds were reported to show improved oral bioavailability and brain penetration [160]. Finally, glycosyl carbamoylalkylidene prodrugs were reported to provide enhanced oral bioavailability of drugs [161].

### Targeting to the colon

Dexamethasone 21-*β*-d-glucoside and prednisolone 21-*β*-d-glucoside were prepared as prodrugs and were investigated for their prospects in treating inflammatory bowel disease [162]. Both glucosides were found to reach the rat lower intestine where they were rapidly hydrolysed, releasing the free steroids. Delivery of dexamethasone via its glucoside to the colon was more specific than that of prednisolone, delivered via its glucoside: nearly 60% of an oral dose of dexamethasone glucoside reached the cecum, whereas less than 15% of prednisolone glucoside reached the cecum. When the free steroids were administered orally, they were almost exclusively absorbed in the small intestine: less than 1% of an oral dose of each reached the cecum. In another study it was found that oral administration of dexamethasone-*β*-d-glucoside led to reduced gross pathological effects (fluid cecal contents, redness, edema, ulcerations) and a significantly lower histopathological score relative to dexamethasone, which was ineffective at controlling the inflammatory response relative to control animals [163]. In contrast to the results with dexamethasone glucoside, the use of prednisolone-21-*O*-*β*-d-glucoside was reported to produce a two-fold increase of oral bioavailability with respect to prednisolone [158]. Similarly, *β*-d-glucoside and *β*-d-galactoside conjugates of 5-aminosalicylic acid were found to be useful to target 5-aminosalicylic acid to the colon. The glycoside was hydrolysed by glycosidase activity from bacterial intestinal flora [164].

### Reduction gastrointestinal disturbances

Sugar prodrugs of a great number of nonsteroidal anti-inflammatory drugs (NSAIDs) have been designed and synthesized. When administered orally, such prodrugs are more soluble than their parent NSAIDs, thus causing less stomach irritation. The rationale behind these sugar prodrugs is that sugar conjugates are expected to exhibit reduced adverse effects particularly in the gastrointestinal (GI) tract. Glycosylated acetaminophen prodrugs were reported to show a higher solubility [165]. An orally administrable prodrug of ketorolac was disclosed as a reversible conjugate with to D-galactose (ketogal) [166]. This prodrug was able to maintain the anti-inflammatory and the analgesic activity of the drug without giving rise to gastric ulcer formation. Ester prodrugs of ibuprofen were synthesized using α-methyl, ethyl and propyl glucopyranoside as promoieties and were tested for their anti-inflammatory, analgesic and ulcerogenic activities [167]. On oral administration the prodrugs did elicit a pharmacological profile quite similar to that of ibuprofen, but, unlike this drug, they displayed reduced gastric ulceration.

Summarized, as the principle challenges in drug administration are the solubility of the active-drug [168, 169] and the permeability of the drug over the intestinal membrane, the structural knowledge of substrates glycoside transporters and hydrolases can be of important advantage to tackle these challenges. We aim that this review focussed on molecular structure, represents a solid simplified bases to start from and to fuel the research in the area of therapeutics and diagnostics.
